# Neurodevelopment Among Publicly Insured Children in the First 5 Years After Infant Heart Surgery

**DOI:** 10.1001/jamanetworkopen.2025.56832

**Published:** 2026-02-04

**Authors:** Daniel O’Meara, Brandi Henson, Caitlin K. Rollins, Kimberlee Gauvreau, Jay G. Berry, Matt Hall, Jane W. Newburger

**Affiliations:** 1 Department of Pediatrics, Boston Children’s Hospital, Boston, Massachusetts; 2 Department of Pediatrics, Harvard Medical School, Boston, Massachusetts; 3 Department of Pediatrics, Emory University School of Medicine, Atlanta, Georgia; 4 Department of Cardiology, Children’s Healthcare of Atlanta, Atlanta, Georgia; 5 Department of Cardiology, Boston Children’s Hospital, Boston, Massachusetts; 6 Department of Psychiatry, Harvard Medical School, Boston, Massachusetts; 7 Department of Neurology, Boston Children’s Hospital, Boston, Massachusetts; 8 Department of Neurology, Harvard Medical School, Boston, Massachusetts; 9 Children’s Hospital Association, Washington, District of Columbia

## Abstract

**Importance:**

Survivors of infant heart surgery for congenital heart disease (CHD) have high risk for neurodevelopmental (ND) disorders; few multistate data report the percentage with ND diagnosis or utilization across health care settings.

**Objective:**

To characterize time to and cumulative prevalence of any ND diagnosis and associated health care utilization in the first 5 years after infant heart surgery for publicly insured children.

**Design, Setting, and Participants:**

This retrospective cohort study included children with CHD with a birth hospitalization and infant cardiac surgery between January 2016 and December 2020. Data were obtained from the Merative MarketScan Medicaid Claims Database of deidentified individual Medicaid enrollees across 12 states. Data were analyzed from December 13, 2022, to October 4, 2024.

**Exposures:**

CHD and infant heart surgery.

**Main Outcomes and Measures:**

Times from index surgery to first ND diagnosis and ND utilization and their cumulative prevalences by 5 years after index surgery.

**Results:**

Among 3147 patients (mean [SD] duration of enrollment, 30.5 [17.4] months), 1686 (53.6%) were male; 619 (19.7%) were Black, 228 (7.2%) were Hispanic, 1051 (33.4%) were White, 145 (4.5%) belonged to additional racial and ethnic groups, and 1108 (35.2%) had missing data on race and ethnicity. Surgery was neonatal (<30 days) in 1518 (48.2%); 709 (22.5%) had a genetic diagnosis; and highest Risk Adjustment for Congenital Heart Surgery (RACHS-2) category was 1 for 945 patients (30.0%), 2 for 915 (29.1%), 3 for 398 (12.9%), 4 for 577 (18.3%), and 5 for 312 (9.9%). The cumulative prevalences of 1 or more ND diagnoses and use of 1 or more forms of ND services were, respectively, 43.5% (95% CI, 42.2%-44.7%) and 80.0% (95% CI, 78.9%-80.9%) at 3 years and 51.7% (95% CI, 50.4%-52.9%) and 82.9% (95% CI, 81.9%-83.8%) at 5 years. Although 1752 (55.7%) had ND screening, fewer had psychological or neuropsychological (207 [6.6%]) or comprehensive developmental (268 [8.5%]) evaluation. Independent risk factors for time to first ND diagnosis were Black race (hazard ratio [HR], 1.14 [95% CI, 1.00-1.30]; *P* = .04) or Hispanic ethnicity (HR, 1.24 [95% CI, 1.02-1.50]; *P* = .03) compared with White race and RACHS-2 category 4 (HR, 1.28 [95% CI, 1.09-1.49]; *P* = .002) or 5 (HR, 1.32 [95% CI, 1.08-1.61]; *P* = .007) compared with category 1. Independent risk factors for time to first ND utilization included RACHS-2 categories 2 (HR, 1.30 [95% CI, 1.06-1.60]; *P* = .01), 4 (HR, 1.63 [95% CI, 1.26-2.11]; *P* < .001), and 5 (HR, 2.25 [95% CI, 1.64-3.10]; *P* < .001) compared with category 1.

**Conclusions and Relevance:**

In this cohort study of publicly insured children who underwent infant heart surgery, the prevalence of ND disorders and utilization of ND services was high. Developmental screening percentages were comparable with those in general pediatric samples, but few children received society-recommended formal ND evaluations.

## Key Points

**Question:**

How prevalent are neurodevelopmental disorders and services within 5 years after infant heart surgery among publicly insured children?

**Findings:**

In this cohort study of 3147 children with an index surgery from 2016 to 2020, the 5-year cumulative prevalence rates of at least 1 neurodevelopmental diagnosis or service were 51.7% and 82.9%, respectively. Brief neurodevelopmental screening occurred in more than half of children, but few underwent psychological or neuropsychological (6.6%) or comprehensive developmental (8.5%) evaluation.

**Meaning:**

In this study, neurodevelopmental diagnoses and services were common after infant heart surgery, but receipt of formal neurodevelopmental assessment was low, suggesting that improved methods are needed to implement society recommendations for universal evaluation in children at high risk.

## Teaser

This cohort study evaluates the rates of neurodevelopmental disorder diagnoses and services among children within 5 years of undergoing infant heart surgery.

## Introduction

Critical congenital heart disease (CCHD), requiring catheter-based or surgical intervention within the first year of life, accounts for nearly 25% of all congenital heart disease (CHD) diagnoses.^1^ Although most children with CCHD now survive to adulthood,^2^ neurodevelopmental impairment remains among their most pervasive comorbidities.^3^ Medical risk factors for adverse neurodevelopmental outcomes include genetic abnormalities, low fetal cerebral oxygen delivery, and perioperative risk factors, such as hypotension and cerebral hypoxia-ischemia.^4,5^ However, social factors, such as low family and neighborhood socioeconomic status (SES), have emerged as the greatest contributors to low neurodevelopmental performance, even in children with the most complex cyanotic heart disease, mirroring the impact of low SES on neurodevelopment in the general population.^6,7,8,9,10,11,12,13,14^ Because access to neurodevelopmental evaluation and intervention may improve the long-term outlook for children with developmental delays,^15,16,17^ there is a critical need for data on neurodevelopmental diagnoses and therapies in children with CCHD who are publicly insured.

In 2012 and 2024, the American Heart Association released scientific statements recommending surveillance, screening, evaluation, reevaluation, and management of developmental disorders in children with CHD.^4,18^ In accordance with this guidance, high-risk children with CHD should undergo routine neurodevelopmental evaluation at ages 12 to 24 months, 3 to 5 years, and again at 11 to 12 years, and those younger than 3 years should receive early intervention (EI). Children with CHD are considered high-risk if they (1) underwent open heart surgery in infancy, (2) have a history of chronic cyanosis, or (3) underwent intervention or hospitalization at younger than 18 years and also have risk factors for neurodevelopmental impairments, including low SES.

Despite wide dissemination of these recommendations, accurate implementation rates for neurodevelopmental evaluation and therapies in diverse patient populations are unknown. Prior reports have focused on survey data of pediatric cardiologist perceptions,^19^ single-center experiences,^20,21^ single-state experience,^22^ or subspecialty centers with existing cardiac neurodevelopmental programs.^23^ However, neurodevelopmental evaluations and interventions may occur outside of the academic medical centers where patients have their cardiac surgery. In the current study, we sought to characterize the times from index surgery to first associated health care utilization and their cumulative prevalences in the first 5 years after infant heart surgery for publicly insured children with CHD.

## Methods

### Study Design, Population, and Setting

We performed a retrospective analysis of children with CCHD who were enrolled in Medicaid, had a birth hospitalization to assess date of birth, and underwent cardiac surgery in the first year of life between January 2016 and December 2020 using the MarketScan Medicaid Claims Database (Merative), an administrative claims database of deidentified individual Medicaid enrollees across 12 states in the United States. Patient health care claims were analyzed for up to 5 years after index cardiac surgery. Continuous Medicaid enrollment was not required. The study was deemed exempt from review and the requirement for informed consent by the institutional review board at Boston Children’s Hospital, since it did not meet criteria for human participant research. This article adheres to the Strengthening the Reporting of Observational Studies in Epidemiology (STROBE) reporting guideline for cohort studies.^24^

We used the *International Classification of Diseases, 10th revision, Clinical Modification* (*ICD-10-CM*) diagnosis codes to distinguish behavioral and neurological diagnoses (eTable 1 in Supplement 1), the *Diagnostic and Statistical Manual of Mental Disorders, 5th Edition* classification system to distinguish neurodevelopmental diagnoses from other behavioral health diagnoses, and version 3 of the pediatric complex chronic conditions (CCC) classification system^25^ to identify comorbid chronic conditions (eTable 2 in Supplement 1). *Current Procedural Terminology* (*CPT*) and Healthcare Common Procedure Coding System (HCPCS) codes were used to assess health service use for psychotherapy, neurodevelopmental testing, EI, neuroimaging, physical therapy (PT), occupational therapy (OT), speech and language therapy (ST), and audiology (eTable 3 in Supplement 1).

Single ventricle status was determined based on the coding of relevant *CPT* or *ICD-10-CM* codes at any time during the study period (eTable 4 in Supplement 1). Hospitalizations and emergency department visits were assessed from the database’s health service data tables. Finally, we evaluated annual neurologic and psychiatric medication utilization using relevant therapeutic classes from the Micromedex Red Book (Merative). The drug classes analyzed included muscle relaxers, anticonvulsants, psychotherapy agents, stimulants, anxiolytic and sedative-hypnotics, antimanic agents, and miscellaneous central nervous system (CNS) agents.

### Study Outcomes

Main study outcomes included times from index surgery in the first year of life to the first occurrence of any (1) neurodevelopmental diagnosis and (2) any neurodevelopmental utilization. Patients who did not experience an event were censored at 5 years after index surgery or their last known follow-up time. Other main outcomes included the cumulative prevalences of any neurodevelopmental diagnosis and any utilization (neurodevelopmental or PT, OT, or ST) by 5 years after index surgery. Secondary outcomes were the percentages of patients with behavioral and neurological diagnoses; types and percentages of diagnostic tests and evaluations (eg, neurodevelopmental testing, neuropsychiatric evaluation, brain magnetic resonance imaging [MRI], computed tomography [CT], or ultrasonography [US]); pharmacy-filled medications for neurodevelopmental, behavioral, and neurological diagnoses; therapies other than medication (eg, PT, OT, ST, and EI); and hospitalizations and emergency department visits.

### Covariates

We extracted patient demographic and clinical characteristics, including race and ethnicity, sex, number of ventricles, Risk Adjustment for Congenital Heart Surgery (RACHS-2) risk category,^26^ neonatal status at time of surgery, heart transplant status, and comorbidities. The database provides optional self-reported race and ethnicity data that are derived from the administrative records of contributing state Medicaid programs. While the underlying state data can provide granular race and ethnicity codes, our database consolidated these into combined race and ethnicity categories of Black, Hispanic, White, additional groups, and missing. Additional groups included all patients whose self-identity was other than Black, Hispanic, or White and those who were multiracial. Race and ethnicity were analyzed because they are social drivers of health that can impact neurodevelopmental outcomes and the services children receive. 

### Statistical Analysis

Patient characteristics and all primary and secondary outcomes were summarized using frequencies and percentages. Percentages of outcomes did not account for differential follow-up of patients within the 5-year period. We used multivariable Cox regression to analyze the association of sociodemographic and medical factors with time to first occurrence of any neurodevelopmental diagnosis or of any neurodevelopmental evaluation, adjusting for risk factors chosen a priori and included in the model regardless of statistical significance.^4^ Patients were censored when their Medicaid enrollment ended. Hazard ratios (HRs) are presented with 95% CIs. To account for attrition bias, inverse probability weighting^27^ was used to estimate the cumulative prevalence of neurodevelopmental diagnosis and evaluation over time; weights were estimated from a logistic regression model containing sex, race, and RACHS-2 risk category. Analyses were performed using SAS version 9.4 (SAS Institute), and *P* < .05 was considered statistically significant.

## Results

### Study Cohort Characteristics

Of 1 293 585 children in the database with a birth hospitalization between 2016 and 2020, 3147 (0.24%) had a RACHS-2 qualifying procedure within the first year of life and were included in our analytic cohort. Demographic characteristics of this cohort are summarized in Table 1. The mean (SD) follow-up time was 2.5 (1.4) years.

**Table 1.** Patient Characteristics

Characteristic	Patients, No. (%) (N = 3147)
Race and ethnicity	
Black	619 (19.7)
Hispanic	228 (7.2)
White	1051 (33.4)
Additional groups^a^	141 (4.5)
Missing	1108 (35.2)
Sex	
Male	1686 (53.6)
Female	1448 (46.0)
Missing	13 (0.4)
No. of ventricles	
2 Ventricles	2018 (64.1)
Single ventricle	1129 (35.9)
RACHS-2 risk category	
1	945 (30.0)
2	915 (29.1)
3	398 (12.6)
4	577 (18.3)
5	312 (9.9)
Neonatal surgery (age ≤30 d)	
No	1629 (51.8)
Yes	1518 (48.2)
Heart transplant	
No	3114 (99.0)
Yes	33 (1.0)
Comorbid chronic conditions^b^	
Neuromuscular	602 (19.1)
Respiratory	653 (20.7)
Kidney	429 (13.6)
Gastrointestinal	1328 (42.2)
Hematology and immunodeficiency	370 (11.8)
Metabolic	224 (7.1)
Congenital or genetic defect	1281 (40.7)
Neonatal	1075 (34.2)
Technology dependent	1505 (47.8)
Transplant	46 (1.5)

Abbreviations: RACHS-2, Risk Adjustment for Congenital Heart Surgery.

^a^ Included all patients whose self-identity was other than Black, Hispanic, or White and those who were multiracial.

^b^ Diagnosed at any time from 2016 to 2021.

### Diagnoses of Neurodevelopmental, Behavioral, and Neurologic Disorders

The percentages of patients with neurodevelopmental, behavioral, and neurologic disorders were calculated from available codes at any time until 5 years following index surgery, not accounting for variable follow-up. Of the 3157 patients in our cohort, 1991 (63.3%) had at least 1 code for a neurodevelopmental, behavioral, or neurologic disorder.

At least 1 neurodevelopmental diagnosis code was found in 1278 patients (40.6%), most commonly communication disorders (791 [25.1%]), motor disorders (585 [18.6%]), and global developmental delays (482 [15.3%]) (eTable 5 in Supplement 1). At least 1 behavioral diagnosis code was identified in 674 patients (21.4%) (eTable 6 in Supplement 1). The most common diagnoses were mental health symptoms (253 [8.0%]), which included irritability, restlessness, and excessive crying, and substance-related and addictive disorders (166 [5.3%]), which included opioid and sedation dependence and withdrawal. There were moderate percentages of feeding and related disorders (122 [3.9%]), trauma- and stress-related disorders (99 [3.1%]), and anxiety disorders (71 [2.3%]). Infrequent diagnoses included obsessive compulsive and related disorders (18 [0.6%]) and depressive disorders (9 [0.3%]).

At least 1 neurologic diagnosis was coded in 1346 patients (42.8%) (eTable 7 in Supplement 1). The most common neurologic conditions in the cohort were brain and spinal cord abnormalities (434 [13.8%]), epilepsy (233 [7.1%]), congenital hypotonia (202 [6.4%]), cerebral palsy and hypoxic-ischemic encephalopathy (124 [3.9%]), cerebrovascular disease (112 [3.6%]), and paralysis or plegia (73 [2.3%]).

### Neurodevelopmental Evaluation and Health Care Utilization

Utilization including neurodevelopmental services, neuroimaging, medications, and hospitalizations was assessed up to 5 years after index surgery, not accounting for variable follow-up. Regarding neurodevelopmental services, developmental screening (a brief questionnaire or checklist completed by a parent to identify concerns that is routinely obtained in general pediatric practice), was completed in 1752 patients (55.7%), and 207 (6.6%) had a brief emotional-behavioral assessment. Far fewer had formal evaluations by qualified developmental or behavioral health specialists, with neuropsychological testing in 207 (6.6%); and developmental test administration in 268 (8.5%). Psychiatric diagnostic interviews, typically completed by licensed mental health professionals, were performed in 101 children (3.2%). Only 83 (2.6%) participated in psychotherapy during the 5-year follow up period. Evaluation and treatment by other specialists were common among patients; 1497 (47.6%) were seen by an occupational or physical therapist, 2406 (76.5%) by a speech and language therapist, 1301 (41.3%) by an audiologist, and 189 (6.0%) received related neurodevelopmental services, including EI services and health-focused interviews. Services related to speech and language pathology included diagnostic procedures and feeding therapy.

**Figure 1.**
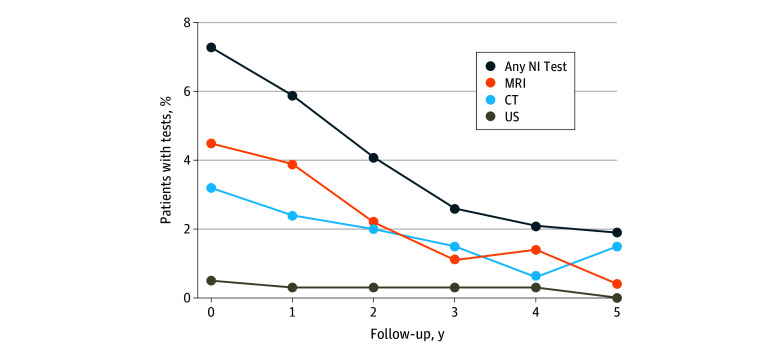
Utilization of Neuroimaging (NI) Tests According to Year of Follow-Up After Index Cardiac Surgery CT indicates computed tomography; MRI, brain magnetic resonance imaging; and US, head ultrasonography.

At least 1 neuroimaging test was performed from index surgery up to 5 years later in 412 patients (13.1%), with 253 (8.0%) having at least 1 MRI, 205 (6.5%) having at least 1 CT, and 30 (1.0%) having at least 1 US (Figure 1). Notably, MRI, CT, and US were performed most often during the first 1 to 2 years of follow-up, with a decline in subsequent years (Figure 1).

At least 1 neurotropic medication was filled from a pharmacy during the 5-year follow-up period for 546 patients (17.3%), with 365 patients (11.6%) receiving anxiolytic or sedative-hypnotics, 226 (7.2%) receiving anticonvulsants, and 105 (3.3%) receiving miscellaneous CNS agents, with stimulants, antipsychotics, and antidepressants filled even less frequently. Notably, atomoxetine is included in the Red Book within miscellaneous CNS agents rather than stimulants.

### Risk Factors for Neurodevelopmental Diagnoses and Evaluations

Risk factors associated with time to first occurrence of diagnosis of at least 1 neurodevelopmental disorder included Hispanic ethnicity (HR, 1.24 [95% CI, 1.02-1.50]; *P* = .03) and Black race (HR, 1.14 [95% CI, 1.00-1.30]; *P* = .04) compared with non-Hispanic White race and ethnicity, as well as RACHS-2 categories 4 (HR, 1.28 [95% CI, 1.09-1.49]; *P* = .002) and 5 (HR, 1.32 [95% CI, 1.08-1.61]; *P* = .007) compared with RACHS-2 category 1 (Table 2). Hematologic and immunodeficiency comorbidities were also associated with time to first diagnosis of at least 1 neurodevelopmental disorder (HR, 0.80 [95% CI, 0.68-0.96]; *P* = .01); the most common conditions in this category of comorbidities were DiGeorge syndrome (103 patients), unspecified immunodeficiency (43 patients), sickle-cell trait (26 patients), and other hemoglobinopathies (14 patients). There were no codes for thromboses or cerebrovascular accidents within this group of comorbidities.

**Table 2.** Univariable and Multivariable Cox Regression Model for First Diagnosis of a Neurodevelopmental Disorder^a^

Characteristic	Univariable		Multivariable	
	HR (95% CI)	*P* value	HR (95% CI)	*P* value
Race and ethnicity				
White	1 [Reference]	NA	1 [Reference]	NA
Black	1.13 (1.00-1.29)	.05	1.14 (1.00-1.30)	.04
Hispanic	1.23 (1.01-1.49)	.03	1.24 (1.02-1.50)	.03
Additional groups	1.25 (1.00-1.55)	.05	1.24 (1.00-1.55)	.05
Missing	1.03 (0.92-1.15)	.59	1.02 (0.91-1.14)	.75
Sex				
Male	1 [Reference]	NA	1 [Reference]	NA
Female	1.04 (0.95-1.14)	.37	1.05 (0.96-1.15)	.29
Missing	5.93 (3.43-10.27)	<.001	5.66 (3.24-9.89)	<.001
No. of ventricles				
2 Ventricles	1 [Reference]	NA	1 [Reference]	NA
Single ventricle	0.96 (0.87-1.06)	.41	0.90 (0.80-1.01)	.08
RACHS-2 risk category				
1	1 [Reference]	NA	1 [Reference]	NA
2	1.10 (0.98-1.24)	.11	1.11 (0.99-1.25)	.08
3	1.09 (0.94-1.27)	.25	1.14 (0.98-1.33)	.09
4	1.13 (0.99-1.29)	.08	1.28 (1.09-1.49)	.002
5	1.15 (0.97-1.36)	.11	1.32 (1.08-1.61)	.007
Comorbid chronic conditions^b^				
Neuromuscular	0.85 (0.74-0.97)	.02	0.87 (0.76-1.00)	.06
Respiratory	0.95 (0.84-1.07)	.38	1.00 (0.89-1.14)	.94
Kidney	1.00 (0.87-1.14)	.95	1.04 (0.90-1.19)	.62
Gastrointestinal	0.91 (0.83-1.01)	.07	0.99 (0.85-1.15)	.90
Hematology and immunodeficiency	0.82 (0.70-0.96)	.01	0.80 (0.68-0.96)	.01
Metabolic	1.01 (0.83-1.24)	.91	1.02 (0.83-1.25)	.87
Neonatal	1.02 (0.93-1.13)	.66	1.04 (0.94-1.15)	.44
Technology dependent	1.02 (0.93-1.13)	.66	0.88 (0.76-1.02)	.10
Transplant	1.07 (0.69-1.64)	.77	1.25 (0.80-1.97)	.32
Genetic	0.97 (0.85-1.11)	.69	1.04 (0.90-1.20)	.59

Abbreviations: HR, hazard ratio; NA, not applicable; RACHS-2, Risk Adjustment for Congenital Heart Surgery.

^a^ Cox proportional hazards regression adjusting for risk factors chosen a priori and included in the model regardless of statistical significance.

^b^ Diagnosed at any time from 2016 to 2021.

Risk factors associated with time to first occurrence of any neurodevelopmental or therapeutic utilization included RACHS-2 categories 2 (HR, 1.30 [95% CI, 1.06-1.60]; *P* = .01), 4 (HR, 1.63 [95% CI, 1.26-2.11]; *P* < .001), and 5 (HR, 2.25 [95% CI, 1.64-3.10]; *P* < .001), in addition to single ventricle (HR, 0.79 [95% CI, 0.64-0.97]; *P* = .02) and technology-dependent comorbidity (HR, 0.67 [95% CI, 0.52-0.86]; *P* = .002) (Table 3). Sex and genetic abnormalities were not independently associated with time to first neurodevelopmental diagnosis or utilization.

**Table 3.** Univariable and Multivariable Cox Regression Model for First Neurodevelopmental or Therapeutic Utilization^a^

Characteristic	Univariable		Multivariable	
	HR (95% CI)	*P* value	HR (95% CI)	*P* value
Race and ethnicity				
White	1 [Reference]	NA	1 [Reference]	NA
Black	1.06 (0.84-1.33)	.65	1.09 (0.86-1.38)	.47
Hispanic	1.23 (0.82-1.86)	.31	1.20 (0.80-1.82)	.38
Additional groups	1.33 (0.88-2.02)	.18	1.29 (0.85-1.97)	.23
Missing	1.13 (0.95-1.35)	.18	1.13 (0.94-1.36)	.19
Sex				
Male	1 [Reference]	NA	1 [Reference]	NA
Female	0.99 (0.84-1.15)	.86	0.99 (0.85-1.16)	.92
Missing	6.39 (1.58-25.90)	.009	6.35 (1.55-26.04)	.01
No. of ventricles				
2 Ventricles	1 [Reference]	NA	1 [Reference]	NA
Single ventricle	0.94 (0.79-1.11)	.45	0.79 (0.64-0.97)	.02
RACHS-2 risk category				
1	1 [Reference]	NA	1 [Reference]	NA
2	1.27 (1.04-1.56)	.02	1.30 (1.06-1.60)	.01
3	1.18 (0.91-1.53)	.21	1.30 (0.99-1.70)	.06
4	1.28 (1.02-1.60)	.04	1.63 (1.26-2.11)	<.001
5	1.59 (1.22-2.08)	.001	2.25 (1.64-3.10)	<.001
Comorbid chronic conditions^b^				
Neuromuscular	0.78 (0.61-1.00)	.05	0.87 (0.67-1.13)	.30
Respiratory	0.95 (0.78-1.15)	.59	1.04 (0.84-1.29)	.71
Kidney	0.94 (0.74-1.20)	.62	1.01 (0.79-1.31)	.91
Gastrointestinal	0.83 (0.69-1.00)	.06	1.05 (0.81-1.36)	.71
Hematology and immunodeficiency	0.79 (0.59-1.06)	.12	0.74 (0.54-1.02)	.07
Metabolic	0.92 (0.64-1.33)	.66	0.86 (0.59-1.26)	.44
Neonatal	1.05 (0.89-1.23)	.57	1.08 (0.92-1.28)	.35
Technology dependent	1.05 (0.89-1.23)	.57	0.67 (0.52-0.86)	.002
Transplant	0.87 (0.39-1.95)	.74	1.14 (0.49-2.66)	.75
Genetic	0.99 (0.77-1.28)	.96	1.14 (0.87-1.50)	.34

Abbreviations: HR, hazard ratio; NA, not applicable; RACHS-2, Risk Adjustment for Congenital Heart Surgery.

^a^ Cox proportional hazards regression adjusting for risk factors chosen a priori and included in the model regardless of statistical significance.

^b^ Diagnosed at any time 2016-2021.

### Cumulative Prevalence of Any Neurodevelopmental Diagnosis or Utilization

**Figure 2.**
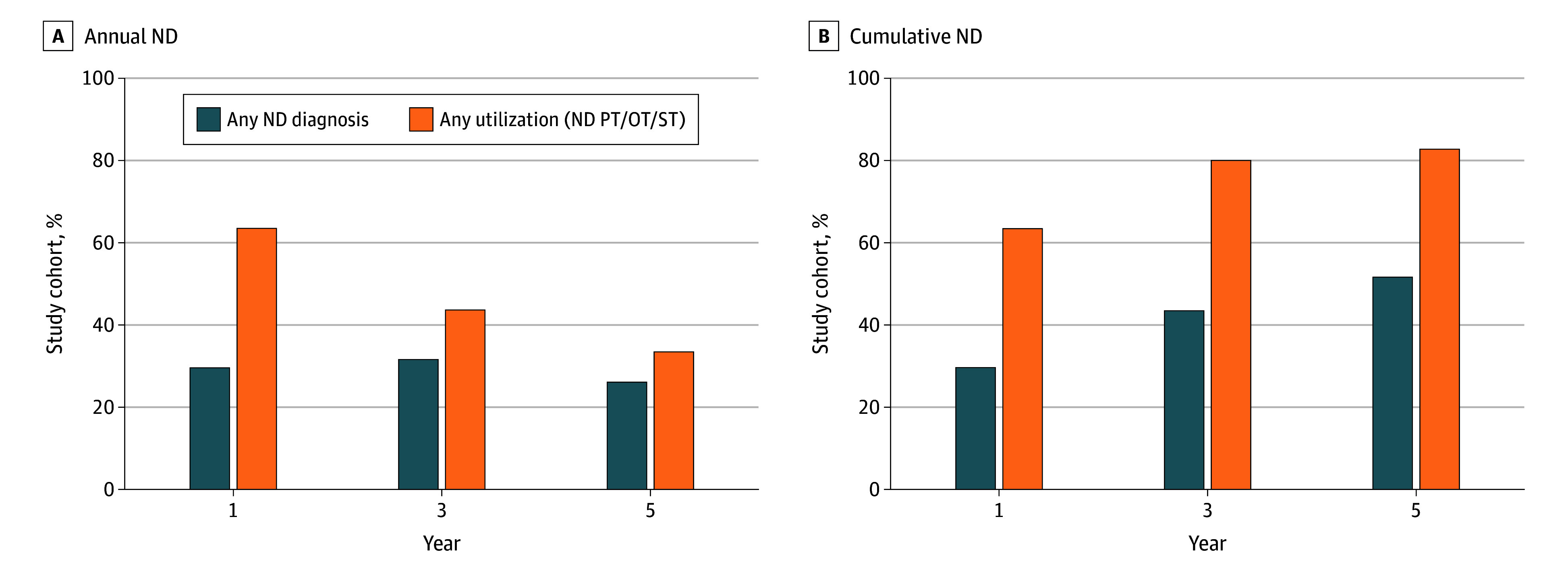
Annual and Cumulative Prevalence of Neurodevelopmental (ND) Diagnosis and Resource Utilization Resources include ND services or physical therapy (PT), occupational therapy (OT), or speech and language therapy (ST). Inverse probability weighting was used to account for variable follow-up.

Using inverse probability weighting to account for variable follow-up, annual prevalences of at least 1 neurodevelopmental diagnosis and utilization of at least 1 form of neurodevelopmental evaluation or therapy in the first year after index surgery were 29.7% and 63.5% respectively. In the third year of follow-up, the percentage receiving a diagnosis increased to 31.6%, with utilization declining to 43.7%. In the fifth year of follow-up, the percentage receiving a diagnosis decreased to 26.1%, with utilization similarly declining to 33.5% (Figure 2A). Using inverse probability weighting again to account for variable follow-up, the cumulative prevalences of at least 1 neurodevelopmental diagnosis and at least 1 form of neurodevelopmental or therapeutic utilization were respectively 43.5% (95% CI, 42.2%-44.7%) and 80.0% (95% CI, 78.9%-80.9%) at 3 years and 51.7% (95% CI, 50.4%-52.9%) and 82.9% (95% CI, 81.9%-83.8%) at 5 years (Figure 2B).

## Discussion

Using the MarketScan Medicaid Claims Database in publicly insured, geographically diverse patients with CCHD and a RACHS-2 qualifying surgery during the first year of life, we found a high prevalence of neurodevelopmental disorders within 5 years of index surgery. Children in our study were more than twice as likely to have received a neurodevelopmental diagnosis by age 5 years than the general population (52% vs 20%).^28^ Risk factors for earlier neurodevelopmental diagnosis included Black or Hispanic race and ethnicity and RACHS-2 categories 4 and 5, reflecting higher surgical complexity. Higher RACHS-2 category was also independently associated with shorter time to first resource utilization, including neurodevelopmental evaluation and services, such as PT, OT, and ST. In contrast, single ventricle and technology dependence were independently associated with longer time to first utilization, possibly because greater medical complexity lowered the priority of neurodevelopmental care or due to residual confounding. We found that utilization of neurodevelopmental services was high in the first year after heart surgery but declined in subsequent years. Our cohort had a higher percentage of screening for developmental concerns (55.7% of patients) than the general population (30.4% between 9 to 35 months),^29^ but few (15.1%) received formal developmental evaluations, potentially limiting their access to targeted neurodevelopmental interventions. These data suggest that further implementation science is needed to improve the incorporation of developmental evaluation for high-risk children with CHD into routine practice.

Our finding of diminishing neurodevelopmental services following the first year after index surgery may be explained by increased access to services like OT, PT, and ST and to the use of diagnostic procedures and interventions that manage feeding difficulties while hospitalized, which are common in young infants after cardiac surgery.^30,31,32,33,34^ This hypothesis is supported by our findings that higher surgical complexity was associated with shorter time to first neurodevelopmental diagnosis and earlier access to assessment and intervention services. Additionally, younger children may have better access to services through EI since cardiac surgery serves as one of the qualifiers in many states, even without a formal developmental diagnosis. By contrast, eligibility for educational or therapeutic services in older children who have aged out of EI requires a documented neurodevelopmental disorder typically obtained through a formal assessment. Indeed, in school-age children, neurodevelopmental evaluation has been linked to subsequent acquisition of more academic services, including individualized education plans, small group academic instruction, and instructional supports.^35^ Thus, regular neurodevelopmental assessment and follow-up are crucial for maintaining access to targeted long-term care for these patients.

Our data build on earlier literature on the implementation of neurodevelopmental assessments in at-risk children with CHD. Because neurodevelopmental practices have been found to vary widely among cardiac surgical centers,^23,36^ the Cardiac Neurodevelopmental Outcome Collaborative (CNOC) published a standardized testing battery for serial neurodevelopmental and behavioral assessment from birth to age 5 years to reduce practice variation and improve quality of care.^37^ This protocol is used at many CNOC centers and results are entered into a central data registry for purposes of quality improvement.^38^ However, the overall implementation rate of follow-up between ages 11 and 30 months at 16 centers was only 29%, with individual rates ranging from 8% to 54%.^23^ Factors enhancing the likelihood of attendance included hospital-initiated scheduling, antenatal diagnosis, greater surgical complexity, longer postoperative length of stay, private insurance, and residence closer to the medical center. A recent analysis of all-payer claims data from the state of Maine from 2015 to 2019 confirmed that only 23% of children younger than 18 years with complex CHD had at least 1 developmental or psychosocial encounter or service.^22^ In this clinical experience, only 28% of encounters for developmental testing took place at hospital-based clinics with subspecialists, and a tiny percentage (1.6%) of children were evaluated at a surgical center, with few predictors of utilization. Our study, which analyzed data from 12 states, bolsters confidence that most young children with public insurance are not undergoing recommended developmental assessments in any location.

### Limitations

We note limitations to this study. Our administrative data were subject to coding errors and to variations in coding practices. Additionally, a single billing claim for a patient encounter was limited to 3 *ICD-10-CM* codes and 1 *CPT* code, limiting a clinician’s ability to document developmental diagnoses that may not be directly related to the encounter and potentially biasing a clinician to submit only those codes that generate the highest revenue. We could not view the types of clinicians who assigned a specific code. Importantly, the database does not account for inpatient care bundling that could occur for children admitted to the hospital, which may affect utilization totals and explain the low percentage of head US. Database restrictions also prevented us from identifying patient states, centers, or zip codes and census tracts, nor could we distinguish between Medicaid or private insurance supplemented with Medicaid. Additionally, nearly one-third of race and ethnicity data were missing in this cohort. These limitations prevented us from further exploring factors associated with neurodevelopmental diagnoses in Black and Hispanic children. Patients in our database had variable years of Medicaid coverage, and those whose index surgeries were in RACHS-2 categories 4 and 5 had lower duration of Medicaid enrollment. Our study database does not provide etiology data for attrition or allow us to determine whether patients had evaluation for or diagnosis of a neurodevelopmental disorder when they no longer had Medicaid insurance. To mitigate bias in estimates due to variable follow-up and attrition, we used inverse probability weighting, with a propensity score developed using sex, race, and RACHS-2 category. Our study design did not allow us to quantify the extent to which first neurodevelopmental diagnosis resulted from optimal compliance with best medical practice, more severe symptoms, or greater patient medical complexity, eg, with longer hospitalization and hence higher risk of acquired brain injury and increased access to health care services. Regardless, prompt diagnosis and intervention are likely to be beneficial across a range of neurodevelopmental risk. Patients in the Merative MarketScan Medicaid Claims Database are all publicly insured, so findings in our study may not be generalizable to those who are privately insured or self-insured. Additionally, our study design did not allow us to evaluate the impact of proposed methods associated with better access to neurodevelopmental and behavioral evaluation, such as hospital-initiated scheduling^23^ or telehealth utilization.^39^

## Conclusions

In this cohort study of publicly insured children with CHD who underwent infant heart surgery, the overall prevalence rates of neurodevelopmental diagnoses and utilization of neurodevelopmental services were high. Percentages of neurodevelopmental screening were higher than those reported in general pediatric samples. However, few children received formal neurodevelopmental evaluation as recommended by the American Heart Association and American Academy of Pediatrics. Future studies should develop and test improved methods to implement recommended neurodevelopmental and behavioral assessment in high-risk children with CHD.

## Notes

Supplement 1. **eTable 1.** Behavioral Health (Including Neurodevelopmental) and Neurological *ICD-10* Codes **eTable 2.** Categories of the Pediatric Complex Comorbid Conditions (CCC) Version 2 Classification System and the Corresponding *ICD-9* and *ICD-10* Diagnosis and Procedure Codes **eTable 3.** *CPT* and HCPCS Codes for Psychotherapy, Neurodevelopmental Testing, Early Intervention, Neuroimaging, Physical Therapy, Occupational Therapy, Speech and Language Therapy, Audiology, and Neurological Testing **eTable 4.** Single Ventricle Procedure and Diagnosis Codes **eTable 5.** Neurodevelopmental Diagnoses at Any Time Until 5 Years Following Index Surgery (Not Accounting for Variable Follow-Up) **eTable 6.** Behavioral Diagnoses at Any Time Until 5 Years Following Index Surgery (Not Accounting for Variable Follow-Up) **eTable 7.** Neurological Diagnoses at Any Time Until 5 Years Following Index Surgery (Not Accounting for Variable Follow-Up)

Supplement 2.
Data Sharing Statement
