# Supplementary material for: Neurodevelopment Among Publicly Insured Children in the First 5 Years After Infant Heart Surgery
Source: JAMA Netw Open. 2026 Feb 4;9(2):e2556832. doi: 10.1001/jamanetworkopen.2025.56832 (PMC12873768; doi:10.1001/jamanetworkopen.2025.56832)
Supplement: Supplement 1. — eTable 1. Behavioral Health (Including Neurodevelopmental) and Neurological ICD-10 Codes eTable 2. Categories of the Pediatric Complex Comorbid Conditions (CCC) Version 2 Classification System and the Corresponding ICD-9 and ICD-10 Diagnosis and Procedure Codes eTable 3. CPT and HCPCS Codes for Psychotherapy, Neurodevelopmental Testing, Early Intervention, Neuroimaging, Physical Therapy, Occupational Therapy, Speech and Language Therapy, Audiology, and Neurological Testing eTable 4. Single Ventricle Procedure and Diagnosis Codes eTable 5. Neurodevelopmental Diagnoses at Any Time Until 5 Years Following Index Surgery (Not Accounting for Variable Follow-Up) eTable 6. Behavioral Diagnoses at Any Time Until 5 Years Following Index Surgery (Not Accounting for Variable Follow-Up) eTable 7. Neurological Diagnoses at Any Time Until 5 Years Following Index Surgery (Not Accounting for Variable Follow-Up) [file jamanetwopen-e2556832-s001.pdf]

Supplemental Online Content

O'Meara D, Henson B, Rollins C, et al. Neurodevelopment among publicly insured children in the first 5 years after infant heart surgery. *JAMA Netw Open.* 2026;9(2):e2556832. doi:10.1001/jamanetworkopen.2025.56832

eTable 1. Behavioral Health (Including Neurodevelopmental) and Neurological *ICD-10* Codes

eTable 2. Categories of the Pediatric Complex Comorbid Conditions (CCC) Version 2 Classification System and the Corresponding *ICD-9* and *ICD-10* Diagnosis and Procedure Codes

eTable 3. *CPT* and HCPCS Codes for Psychotherapy, Neurodevelopmental Testing, Early Intervention, Neuroimaging, Physical Therapy, Occupational Therapy, Speech and Language Therapy, Audiology, and Neurological Testing

eTable 4. Single Ventricle Procedure and Diagnosis Codes

eTable 5. Neurodevelopmental Diagnoses at Any Time Until 5 Years Following Index Surgery (Not Accounting for Variable Follow-Up)

eTable 6. Behavioral Diagnoses at Any Time Until 5 Years Following Index Surgery (Not Accounting for Variable Follow-Up)

eTable 7. Neurological Diagnoses at Any Time Until 5 Years Following Index Surgery (Not Accounting for Variable Follow-Up)

This supplemental material has been provided by the authors to give readers additional information about their work.

© 2026 O'Meara D et al. *JAMA Netw Open*.

eTable 1. Behavioral Health (Including Neurodevelopmental) and Neurological *ICD-10* Codes

**ND ICD10**	**Description**	**Category**
F902	ADHD Combined	ND_ADHD
F900	ADHD Inattentive	ND_ADHD
F901	ADHD Hyperactive/Impulsive	ND_ADHD
F908	Other Specified ADHD	ND_ADHD
F909	Unspecified ADHD	ND_ADHD
F840	Autism Spectrum Disorder	ND_Autism
F802	Language Disorder	ND_Communication
F800	Speech Sound Disorder	ND_Communication
F8081	Childhood Onset Fluency Disorder	ND_Communication
F8082	Social Pragmatic Communication Disorder	ND_Communication
F809	Unspecified Communication Disorder	ND_Communication
F70	Intellectual Disability, Mild	ND_Intellectual_dis
F71	Intellectual Disability, Moderate	ND_Intellectual_dis
F72	Intellectual Disability, Severe	ND_Intellectual_dis
F73	Intellectual Disability, Profound	ND_Intellectual_dis
F79	Unspecified Intellectual Disability	ND_Intellectual_dis
F810	SLD with impairment in reading	ND_Learning_dis
F8181	SLD with impairment in written expression	ND_Learning_dis
F812	SLD with impairment in math	ND_Learning_dis
F82	Developmental Coordination Disorder	ND_Motor_dis
F984	Stereotypic Movement Disorder	ND_Motor_dis
F88	Global Developmental Delay	ND_Neuro_dis
F88	Other Specified Neurodevelopmental Disorder	ND_Neuro_dis
F89	Unspecified Neurodevelopmental Disorder	ND_Neuro_dis
F068	Other Mental Disorder associated with medical history	ND_Other
F952	Tourette's Disorder	ND_Tic_dis
F951	Persistent Motor/Vocal Tic Disorder	ND_Tic_dis
F950	Provisional Tic Disorder	ND_Tic_dis
F958	Otherwise Specified Tic Disorder	ND_Tic_dis
F959	Unspecified Tic Disorder	ND_Tic_dis
**BH ICD10**	**Description**	**Category**
F909	Attention-deficit Hyperactivity Disorder, Unspecified Type	ADHD
F902	Attention-deficit Hyperactivity Disorder, Combined Type	ADHD

© 2026 O'Meara D et al. *JAMA Netw Open*.

F901	Attention-deficit Hyperactivity Disorder, Predominantly Hyperactive Type	ADHD
F900	Attention-deficit Hyperactivity Disorder, Predominantly Inattentive Type	ADHD
T403X1A	Poisoning By Methadone, Accidental (unintentional), Initial Encounter	Accidental or Undetermined Poisoning
T40601A	Poisoning By Unspecified Narcotics, Accidental (unintentional), Initial Encounter	Accidental or Undetermined Poisoning
T40604A	Poisoning By Unspecified Narcotics, Undetermined, Initial Encounter	Accidental or Undetermined Poisoning
T407X1A	Poisoning by cannabis (derivatives), accidental (unintentional), initial encounter	Accidental or Undetermined Poisoning
F419	Anxiety Disorder, Unspecified	Anxiety Disorders
F418	Other Specified Anxiety Disorders	Anxiety Disorders
F411	Generalized Anxiety Disorder	Anxiety Disorders
F930	Separation Anxiety Disorder Of Childhood	Anxiety Disorders
F40298	Other Specified Phobia	Anxiety Disorders
F4010	Social Phobia, Unspecified	Anxiety Disorders
F409	Phobic Anxiety Disorder, Unspecified	Anxiety Disorders
F840	Autistic Disorder	Autism Spectrum Disorder
F849	Pervasive Developmental Disorder, Unspecified	Autism Spectrum Disorder
F848	Other Pervasive Developmental Disorders	Autism Spectrum Disorder
F809	Developmental Disorder Of Speech And Language, Unspecified	Communication Disorders
F8089	Other Developmental Disorders Of Speech And Language	Communication Disorders
F800	Phonological Disorder	Communication Disorders
F804	Speech And Language Development Delay Due To Hearing Loss	Communication Disorders
F8082	Social Pragmatic Communication Disorder	Communication Disorders
F8081	Childhood Onset Fluency Disorder	Communication Disorders
F329	Major Depressive Disorder, Single Episode, Unspecified	Depressive Disorders
F330	Major Depressive Disorder, Recurrent, Mild	Depressive Disorders
F3481	Disruptive Mood Dysregulation Disorder	Depressive Disorders
F88	Other Disorders Of Psychological Development	Developmental Delay or Unspecified Neurodevelopme

© 2026 O'Meara D et al. *JAMA Netw Open*.

F89	Unspecified Disorder Of Psychological Development	Developmental Delay or Unspecified Neurodevelopme
F819	Developmental Disorder Of Scholastic Skills, Unspecified	Developmental Delay or Unspecified Neurodevelopme
F8189	Other Developmental Disorders Of Scholastic Skills	Developmental Delay or Unspecified Neurodevelopme
F919	Conduct Disorder, Unspecified	Disruptive, Impulse Control and Conduct Disorders
F918	Other Conduct Disorders	Disruptive, Impulse Control and Conduct Disorders
F913	Oppositional Defiant Disorder	Disruptive, Impulse Control and Conduct Disorders
F911	Conduct Disorder, Childhood-onset Type	Disruptive, Impulse Control and Conduct Disorders
F639	Impulse Disorder, Unspecified	Disruptive, Impulse Control and Conduct Disorders
F912	Conduct Disorder, Adolescent-onset Type	Disruptive, Impulse Control and Conduct Disorders
F441	Dissociative Fugue	Dissociative Disorders
R159	Full Incontinence Of Feces	Elimination Disorders
F980	Enuresis Not Due To A Substance Or Known Physiological Condition	Elimination Disorders
R150	Incomplete Defecation	Elimination Disorders
F9829	Other Feeding Disorders Of Infancy And Early Childhood	Feeding and Eating Disorders
F983	Pica Of Infancy And Childhood	Feeding and Eating Disorders
F5089	Other Specified Eating Disorder	Feeding and Eating Disorders
F509	Eating Disorder, Unspecified	Feeding and Eating Disorders
F508	Other eating disorders	Feeding and Eating Disorders
P0449	Newborn Affected By Maternal Use Of Other Drugs Of Addiction	Fetal or Newborn Damage Related to Maternal Substa

© 2026 O'Meara D et al. *JAMA Netw Open*.

P961	Neonatal Withdrawal Symptoms From Maternal Use Of Drugs Of Addiction	Fetal or Newborn Damage Related to Maternal Substa
P0441	Newborn Affected By Maternal Use Of Cocaine	Fetal or Newborn Damage Related to Maternal Substa
P043	Newborn Affected By Maternal Use Of Alcohol	Fetal or Newborn Damage Related to Maternal Substa
Q860	Fetal Alcohol Syndrome (dysmorphic)	Fetal or Newborn Damage Related to Maternal Substa
F79	Unspecified Intellectual Disabilities	Intellectual Disability
F72	Severe Intellectual Disabilities	Intellectual Disability
F70	Mild Intellectual Disabilities	Intellectual Disability
F71	Moderate Intellectual Disabilities	Intellectual Disability
F73	Profound Intellectual Disabilities	Intellectual Disability
F78	Other intellectual disabilities	Intellectual Disability
R4183	Borderline Intellectual Functioning	Intellectual Disability
F53	Puerperal psychosis	Maternal Mental Illness or Substance Abuse During
F530	Postpartum Depression	Maternal Mental Illness or Substance Abuse During
O99320	Drug Use Complicating Pregnancy, Unspecified Trimester	Maternal Mental Illness or Substance Abuse During
O99345	Other Mental Disorders Complicating The Puerperium	Maternal Mental Illness or Substance Abuse During
R454	Irritability And Anger	Mental Health Symptom
R451	Restlessness And Agitation	Mental Health Symptom
R4589	Other Symptoms And Signs Involving Emotional State	Mental Health Symptom
R4583	Excessive Crying Of Child, Adolescent Or Adult	Mental Health Symptom
R450	Nervousness	Mental Health Symptom
R464	Slowness And Poor Responsiveness	Mental Health Symptom
R441	Visual Hallucinations	Mental Health Symptom
R4582	Worries	Mental Health Symptom
R4586	Emotional Lability	Mental Health Symptom
R4587	Impulsiveness	Mental Health Symptom
R460	Very Low Level Of Personal Hygiene	Mental Health Symptom
R463	Overactivity	Mental Health Symptom
R443	Hallucinations, Unspecified	Mental Health Symptom
R4681	Obsessive-compulsive Behavior	Mental Health Symptom

© 2026 O'Meara D et al. *JAMA Netw Open*.

R4189	Other Symptoms And Signs Involving Cognitive Functions And Awareness	Miscellaneous
F989	Unspecified Behavioral And Emotional Disorders With Onset Usually Occurring In Childhood And Adolescence	Miscellaneous
F949	Childhood Disorder Of Social Functioning, Unspecified	Miscellaneous
F988	Other Specified Behavioral And Emotional Disorders With Onset Usually Occurring In Childhood And Adolescence	Miscellaneous
R419	Unspecified Symptoms And Signs Involving Cognitive Functions And Awareness	Miscellaneous
F54	Psychological And Behavioral Factors Associated With Disorders Or Diseases Classified Elsewhere	Miscellaneous
R41841	Cognitive Communication Deficit	Miscellaneous
F09	Unspecified Mental Disorder Due To Known Physiological Condition	Miscellaneous
R41840	Attention And Concentration Deficit	Miscellaneous
F99	Mental Disorder, Not Otherwise Specified	Miscellaneous
F39	Unspecified Mood [affective] Disorder	Miscellaneous
F489	Nonpsychotic Mental Disorder, Unspecified	Miscellaneous
F939	Childhood Emotional Disorder, Unspecified	Miscellaneous
R41842	Visuospatial Deficit	Miscellaneous
F82	Specific Developmental Disorder Of Motor Function	Motor Disorders
F984	Stereotyped Movement Disorders	Motor Disorders
F950	Transient Tic Disorder	Motor Disorders
F959	Tic Disorder, Unspecified	Motor Disorders
F05	Delirium Due To Known Physiological Condition	Neurocognitive Disorders
F0280	Dementia In Other Diseases Classified Elsewhere, Unspecified Severity, Without Behavioral Disturbance, Psychotic Disturbance, Mood Disturbance, And Anxiety	Neurocognitive Disorders
F0281	Dementia in other diseases classified elsewhere with behavioral disturbance	Neurocognitive Disorders
F064	Anxiety Disorder Due To Known Physiological Condition	Neurocognitive Disorders
F068	Other Specified Mental Disorders Due To Known Physiological Condition	Neurocognitive Disorders

© 2026 O’Meara D et al. *JAMA Netw Open*.

F0781	Postconcussional Syndrome	Neurocognitive Disorders
F0789	Other Personality And Behavioral Disorders Due To Known Physiological Condition	Neurocognitive Disorders
F4522	Body Dysmorphic Disorder	Obsessive-Compulsive and Related Disorders
F633	Trichotillomania	Obsessive-Compulsive and Related Disorders
F424	Excoriation (skin-picking) Disorder	Obsessive-Compulsive and Related Disorders
F428	Other Obsessive-compulsive Disorder	Obsessive-Compulsive and Related Disorders
F603	Borderline Personality Disorder	Personality Disorders
F209	Schizophrenia, Unspecified	Schizophrenia Spectrum and Other Psychotic Disorde
F22	Delusional Disorders	Schizophrenia Spectrum and Other Psychotic Disorde
F29	Unspecified Psychosis Not Due To A Substance Or Known Physiological Condition	Schizophrenia Spectrum and Other Psychotic Disorde
F521	Sexual Aversion Disorder	Sexuality and Gender Identity Disorders
F514	Sleep Terrors [night Terrors]	Sleep-Wake Disorders
F5104	Psychophysiologic Insomnia	Sleep-Wake Disorders
F5112	Insufficient Sleep Syndrome	Sleep-Wake Disorders
F5101	Primary Insomnia	Sleep-Wake Disorders
F5105	Insomnia Due To Other Mental Disorder	Sleep-Wake Disorders
F513	Sleepwalking [somnambulism]	Sleep-Wake Disorders
F519	Sleep Disorder Not Due To A Substance Or Known Physiological Condition, Unspecified	Sleep-Wake Disorders
F458	Other Somatoform Disorders	Somatic Symptom and Related Disorders
F445	Conversion Disorder With Seizures Or Convulsions	Somatic Symptom and Related Disorders
F4542	Pain Disorder With Related Psychological Factors	Somatic Symptom and Related Disorders
F802	Mixed Receptive-expressive Language Disorder	Specific Learning Disorders
F801	Expressive Language Disorder	Specific Learning Disorders

© 2026 O’Meara D et al. *JAMA Netw Open*.

F8181	Disorder Of Written Expression	Specific Learning Disorders
F810	Specific Reading Disorder	Specific Learning Disorders
K7030	Alcoholic Cirrhosis Of Liver Without Ascites	Substance Abuse-Related Medical Illness
F1123	Opioid Dependence With Withdrawal	Substance-Related and Addictive Disorders
F13239	Sedative, Hypnotic Or Anxiolytic Dependence With Withdrawal, Unspecified	Substance-Related and Addictive Disorders
F1120	Opioid Dependence, Uncomplicated	Substance-Related and Addictive Disorders
F1320	Sedative, Hypnotic Or Anxiolytic Dependence, Uncomplicated	Substance-Related and Addictive Disorders
F1920	Other Psychoactive Substance Dependence, Uncomplicated	Substance-Related and Addictive Disorders
F13230	Sedative, Hypnotic Or Anxiolytic Dependence With Withdrawal, Uncomplicated	Substance-Related and Addictive Disorders
F19939	Other Psychoactive Substance Use, Unspecified With Withdrawal, Unspecified	Substance-Related and Addictive Disorders
F13231	Sedative, Hypnotic Or Anxiolytic Dependence With Withdrawal Delirium	Substance-Related and Addictive Disorders
F17200	Nicotine Dependence, Unspecified, Uncomplicated	Substance-Related and Addictive Disorders
F1910	Other Psychoactive Substance Abuse, Uncomplicated	Substance-Related and Addictive Disorders
F19239	Other Psychoactive Substance Dependence With Withdrawal, Unspecified	Substance-Related and Addictive Disorders
F1129	Opioid Dependence With Unspecified Opioid-induced Disorder	Substance-Related and Addictive Disorders
F1193	Opioid Use, Unspecified With Withdrawal	Substance-Related and Addictive Disorders
F1310	Sedative, Hypnotic Or Anxiolytic Abuse, Uncomplicated	Substance-Related and Addictive Disorders
F1321	Sedative, Hypnotic Or Anxiolytic Dependence, In Remission	Substance-Related and Addictive Disorders
F1510	Other Stimulant Abuse, Uncomplicated	Substance-Related and Addictive Disorders
F1521	Other Stimulant Dependence, In Remission	Substance-Related and Addictive Disorders

© 2026 O'Meara D et al. *JAMA Netw Open*.

F1593	Other Stimulant Use, Unspecified With Withdrawal	Substance-Related and Addictive Disorders
F17210	Nicotine Dependence, Cigarettes, Uncomplicated	Substance-Related and Addictive Disorders
F17290	Nicotine Dependence, Other Tobacco Product, Uncomplicated	Substance-Related and Addictive Disorders
F19230	Other Psychoactive Substance Dependence With Withdrawal, Uncomplicated	Substance-Related and Addictive Disorders
F1990	Other Psychoactive Substance Use, Unspecified, Uncomplicated	Substance-Related and Addictive Disorders
F19921	Other Psychoactive Substance Use, Unspecified With Intoxication With Delirium	Substance-Related and Addictive Disorders
R45851	Suicidal Ideations	Suicide or Self-Injury
T426X2A	Poisoning By Other Antiepileptic And Sedative-hypnotic Drugs, Intentional Self-harm, Initial Encounter	Suicide or Self-Injury
X838XXD	Intentional Self-harm By Other Specified Means, Subsequent Encounter	Suicide or Self-Injury
F430	Acute Stress Reaction	Trauma and Stressor-Related Disorders
F4320	Adjustment Disorder, Unspecified	Trauma and Stressor-Related Disorders
F4325	Adjustment Disorder With Mixed Disturbance Of Emotions And Conduct	Trauma and Stressor-Related Disorders
F4322	Adjustment Disorder With Anxiety	Trauma and Stressor-Related Disorders
F439	Reaction To Severe Stress, Unspecified	Trauma and Stressor-Related Disorders
F4324	Adjustment Disorder With Disturbance Of Conduct	Trauma and Stressor-Related Disorders
F4310	Post-traumatic Stress Disorder, Unspecified	Trauma and Stressor-Related Disorders
F4329	Adjustment Disorder With Other Symptoms	Trauma and Stressor-Related Disorders
F4323	Adjustment Disorder With Mixed Anxiety And Depressed Mood	Trauma and Stressor-Related Disorders
F4312	Post-traumatic Stress Disorder, Chronic	Trauma and Stressor-Related Disorders

© 2026 O'Meara D et al. *JAMA Netw Open*.

F438	Other reactions to severe stress	Trauma and Stressor-Related Disorders
F941	Reactive Attachment Disorder Of Childhood	Trauma and Stressor-Related Disorders

ICD-10 Codes	Neurologic Impairment Category and Specific Diseases
	**Static**
G80, G800, G801, G802, G803, G804, G808, G809, P910, P911, P912, P916, P9160, P9161, P9162, P9163	*Cerebral Palsy and Hypoxic Ischemic Encephalopathy:* Spastic quadriplegic, diplegic, or hemiplegic cerebral palsy; Athetoid cerebral palsy; Ataxic cerebral palsy; Other and unspecified cerebral palsy; Neonatal cerebral ischemia; Acquired periventricular cysts of newborn; Neonatal cerebral leukomalacia; Hypoxic ischemic encephalopathy [HIE] (unspecified, mild, moderate, severe)
G92, G93, G934, G9340, G9341, G9349, G937, G9382, P57, P570, P578, P579, P915, P9181, P91811, P91819, R403	*Encephalopathy*: Toxic encephalopathy; Metabolic encephalopathy; Other and unspecified encephalopathy; Reye's syndrome; Brain death; Kernicterus; Neonatal coma; Neonatal encephalopathy in diseases classified elsewhere or unspecified; Persistent vegetative state
G931, T71111S, T71112S, T71113S, T71114S, T71121S, T71122S, T71123S, T71124S, T71131S, T71132S, T71133S, T71134S, T71141S, T71143S, T71144S, T71151S, T71152S, T71153S, T71154S, T71161S, T71162S, T71163S, T71164S, T71191S, T71192S, T71193S, T71194S, T7120XS, T7121XS, T71221S, T71222S, T71223S, T71224S, T71231S, T71232S, T71233S, T71234S, T7129XS, T719XXS, T744XXS, T751XXS, V9200XS, V9201XS, V9202XS, V9203XS, V9204XS, V9205XS, V9206XS, V9207XS, V9208XS, V9209XS, V9210XS, V9211XS, V9212XS, V9213XS, V9214XS, V9215XS, V9216XS, V9219XS, V9220XS, V9221XS, V9222XS, V9223XS, V9224XS, V9225XS, V9226XS, V9227XS, V9228XS, V9229XS, W65XXXS, W67XXXS, W69XXXS, W73XXXS, W74XXXS, X710XXS, X711XXS, X712XXS, X713XXS, X718XXS, X719XXS, X920XXS, X921XXS, X922XXS, X923XXS, X928XXS, X929XXS, Y210XXS, Y211XXS, Y212XXS, Y213XXS, Y214XXS, Y218XXS, Y219XXS	*Sequelae of Asphyxia and Drowning:* Anoxic brain damage, not elsewhere classified; Asphyxiation due to smothering under pillow [accidental, intentional self-harm, assault, undetermined], sequela; Asphyxiation due to plastic bag [accidental, intentional self-harm, assault, undetermined], sequela; Asphyxiation due to being trapped in bed linens [accidental, intentional self-harm, assault, undetermined], accidental, sequela; Asphyxiation due to smothering under another person's body (in bed), [accidental, assault, undetermined], sequela; Asphyxiation due to smothering in furniture, [accidental, intentional self-harm, assault, undetermined], sequela; Asphyxiation due to hanging, [accidental, intentional self-harm, assault, undetermined], sequela; Asphyxiation due to mechanical threat to breathing due to other causes, [accidental, intentional self-harm, assault, undetermined], sequela; Asphyxiation due to systemic oxygen deficiency due to low oxygen content in ambient air due to unspecified cause, sequela; Asphyxiation due to cave-in or falling earth, sequela; Asphyxiation due to being trapped in a car trunk, [accidental, intentional self-harm, assault, undetermined], sequela; Asphyxiation due to being trapped in a (discarded) refrigerator, [accidental, intentional self-harm, assault, undetermined], sequela;

© 2026 O’Meara D et al. *JAMA Netw Open*.

	Asphyxiation due to being trapped in other low oxygen environment, sequela; Asphyxiation due to unspecified cause, sequela; Shaken infant syndrome, sequela; Unspecified effects of drowning and nonfatal submersion, sequela; Drowning and submersion due to fall off [merchant ship, passenger ship, fishing boat, other powered watercraft, sailboat, canoe or kayak, (nonpowered) inflatable craft, water-skis, other unpowered watercraft, unspecified watercraft] sequela; Drowning and submersion due to being thrown overboard by motion of [merchant ship, passenger ship, fishing boat, other powered watercraft, sailboat, canoe or kayak, (nonpowered) inflatable craft, unspecified watercraft], sequela; Drowning and submersion due to being washed overboard from [merchant ship, passenger ship, fishing boat, other powered watercraft, sailboat, canoe or kayak, (nonpowered) inflatable craft, water-skis, other unpowered water craft, unspecified watercraft], sequela; Accidental drowning and submersion while in [bath-tub, swimming-pool, natural water], sequela; Other specified cause of accidental non-transport drowning and submersion, sequela; Unspecified cause of accidental drowning and submersion, sequela; Intentional self-harm by drowning and submersion [while in bathtub or swimming pool, after jump into swimming pool, in natural water, other, unspecified] sequela; Assault by drowning and submersion [while in bathtub or swimming pool, after push into swimming pool, in natural water, other, unspecified], sequela; Drowning and submersion [while in or after fall into bathtub, while in or after call into swimming pool, in natural water, other, unspecified], undetermined intent, sequela
A50, A500, A5009, A504, A5040, A5041, A5042, A5043, A5045, A5049, A505, A5059, A506, A507, A925, P350, P351, P352	*Congenital Infections:*<br>Congenital syphilis; Early congenital syphilis, symptomatic; Late congenital neurosyphilis [juvenile neurosyphilis]; Late congenital neurosyphilis, unspecified; Late congenital syphilitic meningitis, encephalitis, or polyneuropathy; Juvenile general paresis; Other late congenital neurosyphilis (symptomatic); Late congenital syphilis, latent or unspecified; Zika virus disease; Congenital rubella syndrome; Congenital cytomegalovirus infection; Congenital herpesviral [herpes simplex] infection
	**Anatomic**
G930, G935, G936, G938, G9381, G9389, G939, G94, I780, Q00, Q000, Q001,	*Brain and/or Spinal Cord Abnormalities*:

© 2026 O'Meara D et al. *JAMA Netw Open*.

Q002, Q01, Q010, Q011, Q012, Q018, Q019, Q02, Q04, Q040, Q041, Q042, Q043, Q044, Q045, Q046, Q048, Q049, Q070, Q0700, Q078, Q079, G95, G950, Q06, Q060, Q061, Q062, Q063, Q064, Q068, Q069, Q07, Q761	Cerebral cysts; Compression of brain; Cerebral edema; Temporal sclerosis; Other specified and unspecified disorders of brain; Other disorders of brain diseases classified elsewhere; Hereditary hemorrhagic telangiectasia; Anencephaly; Craniorachischisis; Iniencephaly; Encephalocele (Frontal, nasofrontal, occipital, of other sites, unspecified); Microcephaly; Congenital malformations of corpus callosum; Arhinencephaly; Holoprosencephaly; Other reduction deformities of brain; Septo-optic dysplasia; Megalencephaly; Congenital cerebral cysts; Arnold-Chiari syndrome without spina bifida or hydrocephalus; Other specified and unspecified congenital malformations of brain; Other specified and unspecified congenital malformations of nervous system; Syringomyelia and syringobulbia; Amyelia; Hypoplasia and dysplasia of spinal cord; Diastematomyelia; Hydromyelia; Klippel-Feil syndrome; Other congenital cauda equina malformations; Other and unspecified diseases of spinal cord; Other and unspecified congenital malformations of spinal cord
G91, G910, G911, G912, G913, G914, G918, G919, Q03, Q030, Q031, Q038, Q039, Q0702	*Hydrocephalus:*<br>Congenital hydrocephalus; Malformations of aqueduct of Sylvius; Atresia of Magendie and Luschka; Communicating hydrocephalus; Obstructive hydrocephalus; Idiopathic hydrocephalus; Post traumatic hydrocephalus; Hydrocephalus in diseases classified elsewhere; Other or unspecified hydrocephalus; Arnold-Chiari with hydrocephalus
Q05, Q050, Q051, Q052, Q053, Q054, Q055, Q056, Q057, Q058, Q059, Q0701, Q0703	*Spina Bifida:*<br>Spina bifida, with or without hydrocephalus (cervical, thoracic, lumbar, sacral or unspecified region); Arnold-Chiari syndrome with spina bifida and/or hydrocephalus
Q85, Q850, Q8500, Q8501, Q8502, Q8503, Q8509, Q851, Q858, Q859	*Phakomatoses*:<br>Tuberous sclerosis, Neurofibromatosis (nonmalignant, Type 1 or 2, and unspecified); Schwannomatosis; Other phakomatoses (unspecified, not otherwise classified).
	**Progressive/Movement**
E750, E7500, E7501, E7502, E7509, E751, E7510, E7511, E7519, E7525, E7529, E753, E754	*Leukodystrophies:*<br>GM2 gangliosidosis; Sandhoff disease; Tay-Sachs disease; Other GM2 gangliosidosis; Mucolipidosis IV; Other and unspecified gangliosidosis; Metachromatic leukodystrophy; Neuronal ceroid lipofuscinosis

© 2026 O'Meara D et al. *JAMA Netw Open*.

A810, A8100, A8101, A8109, A811, A812, A818, A8181, A8182, A8183, E752, E7521, E7522, E7523, E7524, E75240, E75241, E75242, E75243, E75248, E75249, E76, E760, E7601, E7602, E7603, E761, E762, E7621, E76210, E76211, E76219, E7622, E7629, E763, E768, E769, F842, G043, G0430, G0431, G0432, G0439, G13, G131, G132, G138, G2582, G31, G310, G3101, G3109, G311, G312, G318, G3181, G3182, G3183, G3185, G3189, G319, G32, G320, G328, G3281, G3289, G901, G903	*Neurodegenerative:*<br>Creutzfeldt-Jakob disease; Subacute sclerosing panencephalitis; Progressive multifocal leukoencephalopathy; Kuru; Gerstmann-Straussler-Scheinker syndrome; Fatal familial insomnia; Other sphingolipidosis; Fabry (-Anderson) disease; Gaucher disease; Krabbe disease; Niemann-Pick disease [type A, B, C, D, other, unspecified]; Disorders of glycosaminoglycan metabolism; Mucopolysaccharidosis, type I; Hurler's syndrome; Hurler-Scheie syndrome; Scheie's syndrome; Mucopolysaccharidosis, type II; Morquio mucopolysaccharidoses [A, B, unspecified]; Sanfilippo mucopolysaccharidoses; Other and unspecified mucopolysaccharidoses; Other disorders of glucosaminoglycan metabolism; Rett's syndrome; Acute necrotizing hemorrhagic encephalopathy; Systemic atrophies primarily affecting central nervous system in diseases classified elsewhere; Stiff-man syndrome; Frontotemporal dementia; Pick's disease; Other frontotemporal dementia; Senile degeneration of brain, not elsewhere classified; Degeneration of nervous system due to alcohol; Alpers disease; Leigh's disease; Dementia with Lewy bodies; Corticobasal degeneration; Other specified or unspecified degenerative diseases of nervous system; Other degenerative disorders of nervous system in diseases classified elsewhere; Familial dysautonomia [Riley-Day]; Multi-system degeneration of the autonomic nervous system
G10, G11, G110, G111, G112, G113, G114, G118, G119, G20, G21, G211, G2111, G2119, G212, G213, G214, G218, G219, G23, G230, G231, G232, G238, G239, G241, G242, G248, G255, G2589, G259, G26	*Movement:*<br>Huntington's disease; Congenital nonprogressive ataxia; Early- or late-onset cerebellar ataxia; Cerebellar ataxia with defective DNA repair; Hereditary spastic paraplegia; Other and unspecified hereditary ataxias; Parkinson's disease; Secondary parkinsonism; Hallervorden-Spatz disease; Progressive supranuclear ophthalmoplegia [Steele-Richardson-Olszewski]; Striatonigral degeneration; Other specified and unspecified degenerative diseases of basal ganglia; Genetic torsion dystonia; Idiopathic nonfamilial dystonia; Other dystonia; Other chorea; Other specified extrapyramidal and movement disorders; Extrapyramidal and movement disorder, unspecified and in diseases classified elsewhere

	Genetic
D821, Q90, Q900, Q901, Q902, Q909, Q91, Q910, Q911, Q912, Q913, Q914, Q915, Q916, Q917, Q92, Q920, Q921, Q922, Q925, Q927, Q928, Q929, Q93, Q930, Q931, Q932, Q933, Q934, Q935, Q937, Q938, Q9381, Q9388, Q9389, Q939, Q95,Q952, Q953, Q955, Q958, Q959, Q970, Q971, Q972, Q973, Q978, Q979, Q98, Q980, Q981, Q983, Q984, Q985, Q986, Q987, Q988,Q989,Q99, Q990, Q991, Q992	Di George's syndrome; Down syndrome; Trisomy 21 (nonmosaicism, mosaicism, translocation, unspecified); Trisomy 18 and Trisomy 13 (nonmosaicism, mosaicism, translocation, unspecified); Whole chromosome trisomy (nonmosaicism, mosaicism); Partial trisomy; Duplications with other complex rearrangements; Triploidy and polyploidy; Other specified or unspecified trisomies and partial trisomies of autosomes; Monosomies and deletions from the autosomes, not elsewhere classified; Whole chromosome monosomy (nonmosaicism or mosaicism); Chromosome replaced with ring, dicentric or isochromosome; Deletion of short arm of chromosome 4 or 5; Deletions with other complex rearrangements; Velo-cardio-facial syndrome; Other deletions of part of a chromosome; Other microdeletions; Other or unspecified deletions from the autosomes; Balanced rearrangements and structural markers, not elsewhere classified; Balanced sex or sex/autosomal rearrangement in abnormal individual; Individual with autosomal fragile site; Other and unspecified balanced rearrangements and structural markers; Karyotype 47, XXX; Female with more than three X chromosomes; Mosaicism, lines with various numbers of X chromosomes; Female with 46, XY karyotype; Klinefelter syndrome karyotype 47, XXY; Klinefelter syndrome, male with more than two X chromosomes; Other male with 46, XX karyotype; Klinefelter syndrome, unspecified; Karyotype 47, XYY; Male with structurally abnormal sex chromosome; Male with sex chromosome mosaicism; Other specified sex chromosome abnormalities (female or male phenotype); Chimera 46, XX/46, XY; 46, XX true hermaphrodite; Fragile X chromosome
	**Metabolic**
E70, E700, E701, E702, E7020, E7021, E7029, E713, E7130, E7131, E71310, E71311, E71312, E71313, E71314, E71318, E7132, E7139, E714, E7140, E7141, E7142, E71440, E715, E7150, E7151, E71510, E71511, E71518, E7152, E71520, E71521, E71522, E71528, E71529, E7153, E7154, E71540, E71541, E71542, E71548, E72, E720, E7200, E7201, E7202, E7203, E7204, E7209, E721, E7210, E7211, E7212, E7219, E722, E7220, E7221, E7222, E7223, E7229, E723, E724, E725, E7250, E7251,	Disorders of aromatic amino-acid metabolism [Classical phenylketonuria; Other hyperphenylalaninemias; Disorders of tyrosine metabolism; Tyrosinemia]; Disorders of fatty-acid metabolism; Disorders of fatty-acid oxidation [Long chain/very long chain, medium chain, or short chain acyl CoA dehydrogenase deficiency; Glutaric aciduria type II; Muscle carnitine palmitoyltransferase deficiency]; Disorders of ketone metabolism; Disorders of carnitine metabolism [Primary carnitine deficiency; Carnitine deficiency due to inborn errors of metabolism; Ruvalcaba-Myhre-Smith syndrome]; Peroxisomal disorders [Disorders of peroxisome biogenesis; Zellweger syndrome; Neonatal adrenoleukodystrophy; X-linked adrenoleukodystrophy

© 2026 O'Meara D et al. *JAMA Netw Open*.

E7252, E7253, E7259, E728, E729, E74, E740, E7400, E7401, E7402, E7403, E7404, E7409, E744, E748, E749, E75, E755, E756, E77, E770, E771, E778, E779, E786, E787, E7870, E7871, E7872, E7879, E788, E7881, E7889, E789, E79, E790, E791, E792, E798, E799, E8309, E851, E884, E8840, E8841, E8842, E8849	(childhood cerebral, adolescent, other, unspecified); Adrenomyeloneuropathy; Rhizomelic chondrodysplasia punctata; Zellweger-like syndrome]; Disorders of amino-acid transport [Cystinuria; Hartnup's disease; Lowe's syndrome; Cystinosis]; Disorders of sulfur-bearing amino-acid metabolism [Homocystinuria; Methylenetetrahydrofolate reductase deficiency]; Disorders of urea cycle metabolism [Argininemia; Arginosuccinic aciduria; Citrullinemia]; Disorders of lysine and hydroxylysine metabolism; Disorders of ornithine metabolism; Disorders of glycine metabolism [Non-ketotic hyperglycinemia; Trimethylaminuria; Hyperoxaluria]; Disorders of amino-acid metabolism; Glycogen storage disease [von Gierke disease; Pompe disease; Cori disease; McArdle disease]; Disorders of pyruvate metabolism and gluconeogenesis; Disorder of carbohydrate metabolism; Disorders of sphingolipid metabolism and other lipid storage disorders; Disorders of glycoprotein metabolism; Defects in post-translational modification of lysosomal enzymes; Defects in glycoprotein degradation; Lipoprotein deficiency; Disorders of bile acid and cholesterol metabolism [Barth syndrome; Smith-Lemli-Opitz syndrome]; Disorder of lipoprotein metabolism [Lipoid dermatoarthritis]; Disorders of purine and pyrimidine metabolism [Hyperuricemia without signs of inflammatory arthritis and tophaceous disease; Lesch-Nyhan syndrome; Myoadenylate deaminase deficiency]; Other disorders of copper metabolism; Neuropathic heredofamilial amyloidosis; Mitochondrial metabolism disorders [MELAS syndrome; MERRF syndrome]
	**Stroke/Hemorrhage**
G45, G450, G451, G452, G453, G454, G458, G459, G46, G460, G461, G462, G463, G464, G465, G466, G467, G468, I63, I630, I6300, I6301, I63011, I63012, I63013, I63019, I6302, I6303, I63031, I63032, I63033, I63039, I6309, I631, I6310, I6311, I63111, I63112, I63113, I63119, I6312, I6313, I63131, I63132, I63133, I63139, I6319, I632, I6320, I6321, I63211, I63212, I63213, I63219, I6322, I6323, I63231, I63232, I63233, I63239, I6329, I633, I6330, I6331, I63311, I63312, I63313, I63319, I6332, I63321, I63322, I63323, I63329, I6333, I63331, I63332, I63333, I63339, I6334,	*Cerebrovascular Disease:*<br>Vertebro-basilar artery syndrome; Carotid artery syndrome (hemispheric); Multiple and bilateral precerebral artery syndromes; Amaurosis fugax; Transient global amnesia; Other and unspecified transient cerebral ischemic attacks and related syndromes; Vascular syndromes of brain in cerebrovascular diseases; Middle, anterior, or posterior cerebral artery syndrome; Brain stem or cerebellar stroke syndrome; Pure motor or pure sensory lacunar syndrome; Other lacunar syndromes; Other vascular syndromes of brain in cerebrovascular diseases; Cerebral infarction; Cerebral infarction due to thrombosis of [vertebral (right, left, bilateral, unspecified), basilar, carotid (right, left, bilateral, unspecified), other or unspecified precerebral] artery; Cerebral infarction due to

I63341, I63342, I63343, I63349, I6339, I634, I6340, I6341, I63411, I63412, I63413, I63419, I6342, I63421, I63422, I63423, I63429, I6343, I63431, I63432, I63433, I63439, I6344, I63441, I63442, I63443, I63449, I6349, I635, I6350, I6351, I63511, I63512, I63513, I63519, I6352, I63521, I63522, I63523, I63529, I6353, I63531, I63532, I63533, I63539, I6354, I63541, I63542, I63543, I63549, I6359, I636, I638, I639, I67, I670, I671,I672, I673, I674, I675, I676, I677, I678, I6781, I6782, I679, I680, I7771, I7774, I7775	embolism of [vertebral (right, left, bilateral, unspecified), basilar, carotid (right, left, bilateral, unspecified), other or unspecified precerebral] artery; Cerebral infarction due to unspecified occlusion or stenosis of [vertebral (right, left, bilateral, unspecified), basilar, carotid (right, left, bilateral, unspecified), other or unspecified precerebral] artery; Cerebral infarction due to thrombosis of [middle (right, left, bilateral, unspecified), anterior (right, left, bilateral, unspecified), posterior (right, left, bilateral, unspecified), cerebellar (right, left, bilateral, unspecified), other or unspecified cerebral] artery; Cerebral infarction due to embolism of [middle (right, left, bilateral, unspecified), anterior (right, left, bilateral, unspecified), posterior (right, left, bilateral, unspecified), cerebellar (right, left, bilateral, unspecified), other or unspecified cerebral] artery; Cerebral infarction due to unspecified occlusion or stenosis of [middle (right, left, bilateral, unspecified), anterior (right, left, bilateral, unspecified), posterior (right, left, bilateral, unspecified), cerebellar (right, left, bilateral, unspecified), other or unspecified cerebral] artery; Cerebral infarction due to cerebral venous thrombosis, nonpyogenic; Other and unspecified cerebral infarction; Dissection of cerebral arteries, nonruptured; Cerebral aneurysm, nonruptured; Cerebral atherosclerosis; Progressive vascular leukoencephalopathy; Hypertensive encephalopathy; Moyamoya disease; Nonpyogenic thrombosis of intracranial venous system; Cerebral arteritis, not elsewhere classified; Acute cerebrovascular insufficiency; Cerebral ischemia; Other and unspecified cerebrovascular disease; Cerebral amyloid angiopathy; Dissection of carotid, vertebral, or other precerebral artery
S061X0S, S061X1S, S061X2S, S061X3S, S061X4S, S061X5S, S061X6, S061X6A, S061X6D, S061X6S, S061X9S, S062X0S, S062X1S, S062X2S, S062X3S, S062X4S, S062X5S, S062X6, S062X6A, S062X6D, S062X6S, S062X9S, S06300S, S06301S, S06302S, S06303S, S06304S, S06305S, S06306, S06306A, S06306D, S06306S, S06309S, S06310S, S06311S, S06312S, S06313S, S06314S, S06315S, S06316, S06316A, S06316D, S06316S, S06319S, S06320S, S06321S, S06322S, S06323S, S06324S, S06325S, S06326, S06326A, S06326D, S06326S, S06329S, S06330S, S06331S, S06332S, S06333S, S06334S, S06335S, S06336, S06336A, S06336D,	*Intracranial Hemorrhage or Injury Sequela:* Traumatic cerebral edema [without loss of consciousness or with loss of consciousness of (of any or unspecified duration)], sequela; Traumatic cerebral edema with loss of consciousness greater than 24 hours without return to pre-existing conscious level with patient surviving, [initial, subsequent, sequela]; Diffuse traumatic brain injury [without loss of consciousness or with loss of consciousness of (of any or unspecified duration)], sequela; Diffuse traumatic brain injury with loss of consciousness greater than 24 hours without return to pre-existing conscious level with patient surviving, [initial, subsequent, sequela]; Unspecified focal traumatic brain injury [without loss of consciousness or with loss of consciousness of (of any or unspecified duration)], sequela; Unspecified focal traumatic brain injury with loss

© 2026 O'Meara D et al. *JAMA Netw Open*.

S06336S, S06339S, S06340S, S06341S, S06342S, S06343S, S06344S, S06345S, S06346, S06346A, S06346D, S06346S, S06349S, S06350S, S06351S, S06352S, S06353S, S06354S, S06355S, S06356, S06356A, S06356D, S06356S, S06359S, S06360S, S06361S, S06362S, S06363S, S06364S, S06365S, S06366, S06366A, S06366D, S06366S, S06369S, S06370S, S06371S, S06372S, S06373S, S06374S, S06375S, S06376, S06376A, S06376D, S06376S, S06379S, S06380S, S06381S, S06382S, S06383S, S06384S, S06385S, S06386, S06386A, S06386D, S06386S, S06389S, S064X0S, S064X1S, S064X2S, S064X3S, S064X4S, S064X5S, S064X6, S064X6A, S064X6D, S064X6S, S064X9S, S065X0S, S065X1S, S065X2S, S065X3S, S065X4S, S065X5S, S065X6, S065X6A, S065X6D, S065X6S, S065X9S, S066X0S, S066X1S, S066X2S, S066X3S, S066X4S, S066X5S, S066X6, S066X6A, S066X6D, S066X6S, S066X9S, S06810S, S06811S, S06812S, S06813S, S06814S, S06815S, S06816, S06816A, S06816D, S06816S, S06819S, S06820S, S06821S, S06822S, S06823S, S06824S, S06825S, S06826, S06826A, S06826D, S06826S, S06829S, S06890S, S06891S, S06892S, S06893S, S06894S, S06895S, S06896, S06896A, S06896D, S06896S, S06899S, S069X0S, S069X1S, S069X2S, S069X3S, S069X4S, S069X5S, S069X6, S069X6A, S069X6D, S069X6S, S069X9S,	of consciousness greater than 24 hours without return to pre-existing conscious level with patient surviving, [initial, subsequent, sequela]; Contusion and laceration of [right, left, unspecified] cerebrum [without loss of consciousness or with loss of consciousness of (of any or unspecified duration)], sequela; Contusion and laceration of [right, left, unspecified] cerebrum with loss of consciousness greater than 24 hours without return to pre-existing conscious level with patient surviving, [initial, subsequent, sequela]; Traumatic hemorrhage of [right, left, unspecified] cerebrum [without loss of consciousness or with loss of consciousness of (of any or unspecified duration)], sequela; Traumatic hemorrhage of [right, left, unspecified] cerebrum with loss of consciousness greater than 24 hours without return to pre-existing conscious level with patient surviving, [initial, subsequent, sequela]; Contusion, laceration, and hemorrhage of cerebellum or brainstem [without loss of consciousness or with loss of consciousness of (of any or unspecified duration)], sequela; Contusion, laceration, and hemorrhage of cerebellum or brainstem with loss of consciousness greater than 24 hours without return to pre-existing conscious level with patient surviving, [initial, subsequent, sequela]; Epidural, traumatic subdural, or traumatic subarachnoid hemorrhage [without loss of consciousness or with loss of consciousness of (of any or unspecified duration)] loss of consciousness, sequela; Epidural, traumatic subdural, or traumatic subarachnoid hemorrhage with loss of consciousness greater than 24 hours without return to pre-existing conscious level with patient surviving, [initial, subsequent, sequela]; Injury of right or left internal carotid artery, intracranial portion, not elsewhere classified [without loss of consciousness or with loss of consciousness of (of any or unspecified duration)], sequela; Injury of right or left internal carotid artery, intracranial portion, not elsewhere classified with loss of consciousness greater than 24 hours without return to pre-existing conscious level with patient surviving, [initial, subsequent, sequela]; Other specified or unspecified intracranial injury [without loss of consciousness or with loss of consciousness of (of any or unspecified duration)], sequela; Other specified or unspecified intracranial injury with loss of consciousness greater than 24 hours without return to pre-existing conscious level with patient surviving, [initial, subsequent, sequela]
	**Peripheral**

© 2026 O'Meara D et al. *JAMA Netw Open*.

G12, G120, G121, G122, G1220, G1221, G1222, G1223, G1224, G1225, G1229, G128, G129	*Anterior Horn Cell Disease:*<br>Spinal muscular atrophy and related syndromes [Infantile spinal muscular atrophy, type I (Werdnig-Hoffman); Other inherited spinal muscular atrophy]; Motor neuron disease [Amyotrophic lateral sclerosis; Progressive bulbar palsy; Primary lateral sclerosis; Familial motor neuron disease; Progressive spinal muscle atrophy]
G71, G710, G711, G7111, G7112, G7113, G7114, G7119, G712, G713, G718, G719, G72, G737, P941, P942	*Muscular Dystrophies and Myopathies:*<br>Primary disorders of muscles; Muscular dystrophy; Myotonic disorders [Myotonic muscular dystrophy; Myotonia congenita; Myotonic chondrodystrophy; Drug induced myotonia]; Congenital myopathies; Mitochondrial myopathy; Myopathy in diseases classified elsewhere; Congenital hyper/hypotonia
G35, G36, G360, G361, G368, G369, G37, G370, G371, G372, G373, G374, G375, G378, G379, G70, G700, G7000, G7001, G701, G702, G708, G7080, G7081, G7089, G709, G73, G731, G733, G951, G9511, G9519	*Myelopathy or Myelitis:*<br>Multiple sclerosis; Neuromyelitis optica [Devic]; Acute and subacute hemorrhagic leukoencephalitis [Hurst]; Other acute disseminated demyelination; Diffuse sclerosis of central nervous system; Central demyelination of corpus callosum; Central pontine myelinolysis; Acute transverse myelitis in demyelinating disease of central nervous system; Subacute necrotizing myelitis of central nervous system; Concentric sclerosis [Balo] of central nervous system; Other demyelinating diseases of central nervous system; Myasthenia gravis (with or without acute exacerbation); Toxic myoneural disorders; Congenital and developmental myasthenia; Lambert-Eaton syndrome; Lambert-Eaton syndrome in neoplastic disease; Other myoneural disorders; Myasthenic syndromes in other diseases classified elsewhere; Vascular myelopathies; Acute infarction of spinal cord (embolic or nonembolic); Other vascular myelopathies
A80, A800, A801, A802, A803, A8030, A8039, A804, A809, B91, G14, G041, G723,<br>G82, G822, G8220, G8221, G8222, G825, G8250, G8251, G8252, G8253, G8254, G83, G834, G835, G838, G8381, G8382, G8383, G8389, G839, S140XXS, S14101S, S14102S, S14103S, S14104S, S14105S, S14106S, S14107S ,S14108S, S14109S, S14111S, S14112S, S14113S, S14114S, S14115S, S14116S, S14117S, S14118S, S14119S, S14121S, S14122S, S14123S, S14124S, S14125S, S14126S, S14127S,	*Paralytic Syndromes (including spinal cord injury sequela):*<br>Acute paralytic poliomyelitis (wild virus, imported or indigenous; other and unspecified); Sequelae of poliomyelitis; Postpolio syndrome; Tropical spastic paraplegia; Periodic paralysis; Paraplegia (paraparesis) and quadriplegia (quadriparesis) [complete and incomplete]; Cauda equina syndrome; Locked-in state; Brown-Sequard syndrome; Anterior cord syndrome; Posterior cord syndrome; Concussion and edema of cervical spinal cord, sequela; Unspecified injury at (C1, C2, C3, C4, C5, C6, C7, C8 or unspecified level) of cervical spinal cord, sequela; Complete lesion at (C1, C2, C3, C4, C5, C6, C7, C8 or unspecified level) of cervical spinal cord, sequela; Central

S14128S, S14129S, S14131S, S14132S, S14133S, S14134S, S14135S, S14136S, S14137S, S14138S, S14139S, S14141S, S14142S, S14143S, S14144S, S14145S, S14146S, S14147S, S14148S, S14149S, S14151S, S14152S, S14153S, S14154S, S14155S, S14156S, S14157S, S14158S, S14159S, S145XXS, S240XXS, S24101S, S24102S, S24103S, S24104S, S24109S, S24111S, S24112S, S24113S, S24114S, S24119S, S24131S, S24132S, S24133S, S24134S, S24139S, S24141S, S24142S, S24143S, S24144S, S24149S, S24151S, S24152S, S24153S, S24154S, S24159S, S244XXS, S3401XS, S3402XS, S34101S, S34102S, S34103S, S34104S, S34105S, S34109S, S34111S, S34112S, S34113S, S34114S, S34115S, S34119S, S34121S, S34122S, S34123S, S34124S, S34125S, S34129S, S34131S, S34132S, S34139S, S343XXS, S344XXS, S345XXS	cord syndrome at (C1, C2, C3, C4, C5, C6, C7, C8 or unspecified level) of cervical spinal cord, sequela; Anterior cord syndrome at (C1, C2, C3, C4, C5, C6, C7, C8 or unspecified level) of cervical spinal cord, sequela; Brown-Sequard syndrome at (C1, C2, C3, C4, C5, C6, C7, C8 or unspecified level) of cervical spinal cord, sequela; Other incomplete lesion at (C1, C2, C3, C4, C5, C6, C7, C8 or unspecified level) of cervical spinal cord, sequela; Injury of cervical sympathetic nerves, sequela; Concussion and edema of thoracic spinal cord, sequela; Unspecified injury at (T1, T2-T6, T7-10, T11-12 level or unspecified level) of thoracic spinal cord, sequela; Complete lesion at (T1, T2-T6, T7-10, T11-12 level or unspecified level) of thoracic spinal cord, sequela; Anterior cord syndrome at (T1, T2-T6, T7-10, T11-12 level or unspecified level) l of thoracic spinal cord, sequela; Brown-Sequard syndrome at (T1, T2-T6, T7-10, T11-12 level or unspecified level) of thoracic spinal cord, sequela; Other incomplete lesion at (T1, T2-T6, T7-10, T11-12 level or unspecified level) of thoracic spinal cord, sequela; Injury of thoracic sympathetic nervous system, sequela; Concussion and edema of lumbar spinal cord, sequela; Unspecified injury to (L1, L2, L3, L4, L5 or unspecified level) of lumbar spinal cord, sequela; Complete lesion of (L1, L2, L3, L4, L5 or unspecified level) of lumbar spinal cord, sequela; Incomplete lesion of (L1, L2, L3, L4, L5 or unspecified level) of lumbar spinal cord, sequela; Concussion and edema of sacral spinal cord, sequela; Complete lesion of sacral spinal cord, sequela; Incomplete lesion of sacral spinal cord, sequela; Unspecified injury to sacral spinal cord, sequela; Injury of cauda equina, sequela; Injury of lumbosacral plexus, sequela; Injury of lumbar, sacral and pelvic sympathetic nerves, sequela
G81, G810, G8100, G8101, G8102, G8103, G8104, G811, G8110, G8111, G8112, G8113, G8114, G819, G8190, G8191, G8192, G8193, G8194, G830, G831, G8310, G8311, G8312, G8313, G8314, G832, G8320, G8321, G8322, G8323, G8324, G833, G8330, G8331, G8332, G8333, G8334, I6903, I69031, I69032, I69033, I69034, I69039, I6904, I69041, I69042,I69043, I69044, I69049, I6905, I69051, I69052, I69053, I69054, I69059, I6906, I69061, I69062, I69063, I69064, I69065, I69069, I6913, I69131, I69132, I69133, I69134, I69139, I6914,	*Plegias (including cerebrovascular disease and brain injury sequela)*:<br>Flaccid hemiplegia [affecting right or left (dominant or nondominant), or unspecified side]; Spastic hemiplegia [affecting right or left (dominant or nondominant), or unspecified side]; Hemiplegia, unspecified [affecting right or left (dominant or nondominant), or unspecified side]; Diplegia of upper limbs; Monoplegia of lower or upper limb [affecting right or left (dominant or nondominant), or unspecified side]; Monoplegia, unspecified [affecting right or left (dominant or nondominant), or unspecified side]; Monoplegia of upper or lower limb following nontraumatic subarachnoid hemorrhage [affecting right or left (dominant or nondominant), or unspecified side];

I69141, I69142, I69143, I69144, I69149, I69151, I69152, I69153, I69154, I69159, I6916, I69161, I69162, I69163, I69164, I69165, I69169, I6923, I69231, I69232, I69233, I69234, I69239, I6924, I69241, I69242, I69243, I69244, I69249, I6925, I69251, I69252, I69253, I69254, I69259, I6926, I69261, I69262, I69263, I69264, I69265, I69269, I6933, I69331, I69332, I69333, I69334, I69339, I6934, I69341, I69342, I69343, I69344, I69349, I6935, I69351, I69352, I69353, I69354, I69359, I6936, I69361, I69362, I69363, I69364, I69365, I69369, I6983, I69831, I69832, I69833, I69834, I69839, I6984, I69841, I69842, I69843, I69844, I69849, I6985, I69851, I69852, I69853, I69854, I69859, I6986, I69861, I69862, I69863, I69864, I69865, I69869, I6993, I69931, I69932, I69933, I69934, I69939, I6994, I69941, I69942, I69943, I69944, I69949, I6995, I69951, I69952, I69953, I69954, I69959, I6996, I69961, I69962, I69963, I69964, I69965, I69969	Hemiplegia and hemiparesis following nontraumatic subarachnoid hemorrhage [affecting right or left (dominant or nondominant), or unspecified side]; Other paralytic syndrome following nontraumatic subarachnoid hemorrhage [affecting right or left (dominant or nondominant), bilateral, or unspecified side]; Monoplegia of upper or lower limb following nontraumatic intracerebral hemorrhage [affecting right or left (dominant or nondominant), or unspecified side]; Hemiplegia and hemiparesis following nontraumatic intracerebral hemorrhage [affecting right or left, (dominant or nondominant), or unspecified side]; Other paralytic syndrome following nontraumatic intracerebral hemorrhage [affecting right or left, (dominant or nondominant), bilateral, or unspecified side]; Monoplegia of upper or lower limb following other nontraumatic intracranial hemorrhage [affecting right or left, (dominant or nondominant), or unspecified side]; Hemiplegia and hemiparesis following other nontraumatic intracranial hemorrhage [affecting right or left (dominant or nondominant), or unspecified side]; Other paralytic syndrome following other nontraumatic intracranial hemorrhage [affecting right or left (dominant or nondominant), bilateral, or unspecified side]; Monoplegia of upper or lower limb following cerebral infarction [affecting right or left (dominant or nondominant), or unspecified side]; Hemiplegia and hemiparesis following cerebral infarction [affecting right or left (dominant or nondominant), or unspecified side]; Other paralytic syndrome following cerebral infarction [affecting right or left (dominant or nondominant), bilateral or unspecified side]; Monoplegia of upper or lower limb following other cerebrovascular disease [affecting right or left (dominant or nondominant), or unspecified side]; Hemiplegia and hemiparesis following other cerebrovascular disease [affecting right or left (dominant or nondominant), or unspecified side]; Other paralytic syndrome following other cerebrovascular disease [affecting right or left (dominant or nondominant), bilateral, or unspecified side]; Monoplegia of upper or lower limb following unspecified cerebrovascular disease [affecting right or left (dominant or nondominant), or unspecified side]; Hemiplegia and hemiparesis following unspecified cerebrovascular disease [affecting right or left (dominant or nondominant), or unspecified side]; Other paralytic syndrome following unspecified cerebrovascular disease [affecting right or left (dominant or nondominant) bilateral, or unspecified side]

© 2026 O'Meara D et al. *JAMA Netw Open*.

G130, G540, G541, G600, G601, G602, G603, G608, G609, G611, G6182, G6189, G619, G6289, G629, G63, G650, G651, G652	*Neuropathies*:<br>Paraneoplastic neuromyopathy and neuropathy; Brachial plexus disorders; Lumbosacral plexus disorders; Hereditary motor and sensory neuropathy; Refsum's disease; Neuropathy in association with hereditary ataxia; Idiopathic progressive neuropathy; Other and unspecified hereditary and idiopathic neuropathies; Serum neuropathy; Multifocal motor neuropathy; Other and unspecified inflammatory polyneuropathies; Inflammatory polyneuropathy, unspecified; Other specified and unspecified polyneuropathies; Polyneuropathy in diseases classified elsewhere; Sequelae of Guillain-Barre syndrome; Sequelae of other inflammatory polyneuropathy; Sequelae of toxic polyneuropathy
	**Epilepsy**
G40, G400, G4000, G40001, G40009, G4001, G40011, G40019, G401, G4010, G40101, G40109, G4011, G40111, G40119, G402, G4020, G40201, G40209, G4021, G40211, G40219, G403, G4030, G40301, G40309, G4031, G40311, G40319, G404, G4040, G40401, G40409, G4041, G40411, G40419, G405, G4050, G40501, G40509, G408, G4080, G40801, G40802, G40803, G40804, G4081, G40811, G40812, G40813, G40814, G4082, G40821, G40822, G40823, G40824, G4089, G409, G4090, G40901, G40909, G4091, G40911, G40919, G40A, G40A0, G40A01, G40A09, G40A1, G40A11, G40A19, G40B, G40B0, G40B01, G40B09, G40B1, G40B11, G40B19	Localization-related (focal, partial) idiopathic epilepsy and epileptic syndromes with seizures of localized onset (intractable or not intractable, with or without status epilepticus); Localization-related (focal, partial) symptomatic epilepsy and epileptic syndromes with simple or complex partial seizures (intractable or not intractable, with or without status epilepticus); Generalized idiopathic epilepsy and epileptic syndromes (intractable or not intractable, with or without status epilepticus); Other generalized epilepsy and epileptic syndromes (intractable or not intractable, with or without status epilepticus); Epileptic seizures related to external causes (not intractable, with or without status epilepticus); Other epilepsy (intractable or not intractable, with or without status epilepticus); Lennox-Gastaut syndrome (intractable or not intractable, with or without status epilepticus); Epileptic spasms (intractable or not intractable, with or without status epilepticus); Epilepsy, unspecified (intractable or not intractable, with or without status epilepticus); Absence epileptic syndrome (intractable or not intractable, with or without status epilepticus); Juvenile myoclonic epilepsy [impulsive petit mal] (intractable or not intractable, with or without status epilepticus)

© 2026 O'Meara D et al. *JAMA Netw Open*.

eTable 2. Categories of the Pediatric Complex Comorbid Conditions (CCC) Version 2 Classification System and the Corresponding *ICD-9* and *ICD-10* Diagnosis and Procedure Codes

Categories	Subcategories	ICD-9	ICD-10
**Neurologic and Neuromuscular**	Brain and spinal cord malformations	740.0-742.9	Q00-Q07, G90.1
	Mental retardation	318.0-318.2	F71-F73
	CNS degeneration and diseases	330.0-330.9, 334, 335.0-335.9, 331.1, 331.11, 331.19, 331.4, 331.8, 331.89, 331.9, 333.2, 336.1, 336.8, 337.9, 759.5	E75.0, E75.1, E75.2, E75.4, F84.2, G11.1-G11.4, G11.8, G11.9, G12.0-G12.2, G12.8, G12.9, G31.01, G31.09, G31.8, G31.89, G32.89, G93.8, G93.9, G94, G91.1, G31.9, G25.3, G95.19, G95.89, G90.9, Q85.1
	Infantile cerebral palsy	343.0-343.9	G80
	Epilepsy	345.01, 345.11, 345.3, 345.41, 345.61, 345.71, 345.81, 345.91	G40.311, G40.301, G40.211, G40.219, G40.411, G40.419, G40.111, G40.119, G40.804, G40.911, G40.919
	Other disorders of CNS	341.8, 342.90, 344.0, 344.81, 344.9, 348.1, 348.4, 780.03, 01.52, 01.53	G37.1, G37.2, G37.8, G81.90, G82.90, G82.50-G82.54, G83.5, G83.9, G93.1, G93.5, R40.3, 0016070, 0016071, 0016072, 0016073, 0016074, 0016075, 0016076, 0016077, 0016078, 001607B, 0016370, 0016371,

© 2026 O'Meara D et al. *JAMA Netw Open*.

Categories	Subcategories	ICD-9	ICD-10
			0016372, 0016373, 0016374, 0016375, 0016376, 0016377, 0016378, 001637B, 001U074, 001U076, 001U077, 001U079, 001U374, 001U376, 001U377, 001U379, 00B70ZZ, 00B73ZZ, 00B74ZZ, 00T70ZZ, 00T73ZZ, 00T74ZZ
	Occlusion of cerebral arteries	434.01, 434.91	I63.30, I63.50
	Muscular dystrophies and myopathies	359.0-359.3	G71, G72
	Movement diseases	332.0, 332.1, 333.0, 333.2,33.4, 333.5, 333.7, 333.9	G10, G20, G21.0, G21.11, G21.19, G21.8, G23.0-G23.2, G23.8, G24.02, G24.8, G25.3-G25.5, G25.81-G25.83, G25.89, G25.9, G80.3
	Devices	996.2, 996.63, V45.2, V53.01, V53.02, 02.2, 02.21, 02.22, 02.3, 02.31, 02.32, 02.33, 02.34, 02.35, 02.39, 02.4, 02.41, 02.42, 02.93, 03.7, 03.71, 03.72. 03.79, 03.93, 03.97, 04.92	T85.09XA, T85.190A, T85.192A, T85.199A, T85.79XA, Z98.2, Z45.41, Z45.42, 00160J0, 00160J1, 00160J2, 00160J3, 00160J4, 00160J5, 00160J6, 00160J7,

© 2026 O'Meara D et al. *JAMA Netw Open*.

Categories	Subcategories	ICD-9	ICD-10
			00160J8, 00160JB, 00160K0, 00160K1, 00160K2, 00160K3, 00160K4, 00160K5, 00160K6, 00160K7, 00160K8, 00160KB, 00163J0, 00163J1, 00163J2, 00163J3, 00163J4, 00163J5, 00163J6, 00163J7, 00163J8, 00163JB, 00163K0, 00163K1, 00163K2, 00163K3, 00163K4, 00163K5, 00163K6, 00163K7, 00163K8, 00163KB, 001U0J4, 001U0J6, 001U0J7, 001U0J9, 001U0K4, 001U0K6, 001U0K7, 001U0K9, 001U3J4, 001U3J6, 001U3J7, 001U3J9, 001U3K4, 001U3K6, 001U3K7, 001U3K9, 009600Z, 009630Z, 009640Z,

© 2026 O’Meara D et al. *JAMA Netw Open*.

Categories	Subcategories	ICD-9	ICD-10
			00H00MZ, 00H03MZ, 00H04MZ, 00H60MZ, 00H63MZ, 00H64MZ, 00HE0MZ, 00HE3MZ, 00HE4MZ, 00HU0MZ, 00HU3MZ, 00HU4MZ, 00HV0MZ, 00HV3MZ, 00HV4MZ, 00W60JZ, 00W63JZ, 00W64JZ, 00WU0JZ, 00WU3JZ, 00WU4JZ, 01HY0MZ, 01HY3MZ, 01HY4MZ, 0DH60MZ, 0DH63MZ, 0DH64MZ, 0W110J9, 0W110JB, 0W110JG, 0W110JJ, 3E1Q38X, 3E1Q38Z
	Transplantation	N/A	N/A
**Cardiovascular**	Heart and great vessel malformations	745.0-745.3, 745.60-745.69, 746, 747.1-747.49, 747.81, 747.89, 35.8, 35.81, 35.82, 35.83, 35.84	Q20, Q21.2-Q24, Q25.1-Q26, Q28.2, Q28.3, Q28.9, 02170ZP, 02170ZQ, 02170ZR, 02BK0ZZ, 02LR0ZT, 02LS0ZZ, 02LT0ZZ, 02NH0ZZ,

© 2026 O'Meara D et al. *JAMA Netw Open*.

Categories	Subcategories	ICD-9	ICD-10
			02RK0JZ, 02RL0JZ, 02RM0JZ, 02RP0JZ, 02RQ07Z, 02RQ0JZ, 02RR07Z, 02RR0JZ, 02SP0ZZ, 02SW0ZZ, 02U70JZ, 02UA0JZ, 02UA3JZ, 02UA4JZ, 02VR0ZT, 02WA0JZ
	Endocardium diseases	424.0, 424.2, 424.3	I34.0, I34.8, I36.0, I36.8, I37.0, I37.8
	Cardiomyopathies	425.0-425.4, 425.8, 429.1	I42, I43, I51.5
	Conduction disorder	426.0-427.4	I44, I45, I47, I48, I49.0
	Dysrhythmias	427.6-427.9	I49.1-I49.5, I49.8, I49.9, R00.1
	Other	416.1, 416.8, 416.9, 428.0, 429.3, 428.83, 433.11, V45.81	I27.0, I27.1, I27.2, I27.81, I27.89, I27.9, I50.9, I51.7, I51.81, I63.139, I63.239, Z95.1
	Devices	996.0, 996.1, 996.61, 996.62, V43.3, V45.0, V53.31, V53.32, V53.39, 00.50, 00.51, 00.53, 00.54, 00.55, 00.57, 17.51, 17.52, 37.41, 37.52, 37.53, 37.54, 37.55, 37.6, 37.60, 37.61, 37.63, 37.65, 37.66, 37.67, 37.68, 37.7, 37.71, 37.72, 37.74, 37.76, 37.79, 37.8, 37.80, 37.81, 37.82, 37.83, 37.85, 37.86, 37.87, 37.89, 37.94, 37.95, 37.96, 37.97, 37.98, 39.81, 39.82, 39.83, 39.84, 39.85, 89.46, 89.47, 89.48, 89.49	T82.519A, T82.529A, T82.539A, T82.599A, T82.110A, T82.111A, T82.120A, T82.121A, T82.190A, T82.191A, T82.01XA, T82.02XA, T82.03XA, T82.09XA, T82.211A, T82.212A, T82.213A,

© 2026 O’Meara D et al. *JAMA Netw Open*.

Categories	Subcategories	ICD-9	ICD-10
			T82.218A, T82.221A, T82.222A, T82.223A, T82.228A, T82.518A, T82.528A, T82.538A, T82.598A, T82.6XXA, T82.7XXA, Z95.0, Z95.2, Z95.3, Z95.810-Z95.812, Z95.818, Z45.010, Z45.018, Z45.02, Z45.09, Z95.9, 02H40JZ, 02H40KZ, 02H43JZ, 02H44JZ, 02H44KZ, 02H60JZ, 02H60KZ, 02H63JZ, 02H63KZ, 02H63MZ, 02H64JZ, 02H64KZ, 02H70KZ, 02H73JZ, 02H73KZ, 02H73MZ, 02H74KZ, 02HA0QZ, 02HA0RS, 02HA0RZ, 02HA3QZ, 02HA3RS, 02HA4QZ, 02HA4RS, 02HK0JZ, 02HK0KZ, 02HK3JZ, 02HK3KZ, 02HK3MZ,

© 2026 O'Meara D et al. *JAMA Netw Open*.

Categories	Subcategories	ICD-9	ICD-10
			02HK4JZ, 02HK4KZ, 02HL0JZ, 02HL0KZ, 02HL0MZ, 02HL3JZ, 02HL3KZ, 02HL3MZ, 02HL4JZ, 02HL4KZ, 02HL4MZ, 02HN0JZ, 02HN0KZ, 02HN0MZ, 02HN3JZ, 02HN3KZ, 02HN3MZ, 02HN4JZ, 02HN4KZ, 02HN4MZ, 02WA0QZ, 02WA0RZ, 02WA3QZ, 02WA3RZ, 02WA4QZ, 02WA4RZ, 03HK0MZ, 03HK3MZ, 03HK4MZ, 03HL0MZ, 03HL3MZ, 03HL4MZ, 03WY0MZ, 03WY3MZ, 03WY4MZ, 0JH600Z, 0JH605Z, 0JH606Z, 0JH607Z, 0JH608Z, 0JH609Z, 0JH60AZ, 0JH60MZ, 0JH60PZ, 0JH630Z,

© 2026 O'Meara D et al. *JAMA Netw Open*.

Categories	Subcategories	ICD-9	ICD-10
			0JH635Z, 0JH636Z, 0JH637Z, 0JH638Z, 0JH639Z, 0JH63AZ, 0JH63MZ, 0JH63PZ, 0JH70MZ, 0JH73MZ, 0JH800Z, 0JH805Z, 0JH806Z, 0JH807Z, 0JH808Z, 0JH809Z, 0JH80AZ, 0JH80MZ, 0JH80PZ, 0JH830Z, 0JH835Z, 0JH836Z, 0JH837Z, 0JH838Z, 0JH839Z, 0JH83AZ, 0JH83MZ, 0JH83PZ, 0JWT0MZ, 0JWT0PZ, 0JWT3MZ, 0JWT3PZ, 0JWTXMZ, 4B02XSZ, 4B02XTZ, 5A02110, 5A02116, 5A0211D, 5A02210, 5A02216, 5A0221D
	Transplantation	996.83,V42.1, V42.2, V43.2, 37.5, 37.51	T86.20-T86.22, Z94.1, 02YA0Z0, 02YA0Z1, 02YA0Z2

© 2026 O’Meara D et al. *JAMA Netw Open*.

Categories	Subcategories	ICD-9	ICD-10
**Respiratory**	Respiratory malformations	748.0-748.9	Q30-Q34, P280
	Chronic respiratory diseases	327.25, 416.2, 516.3, 516.31, 518.84, 770.4, V45.76	G47.35, I27.82, I43, J84.112, J96.20, Z90.2
	Cystic fibrosis	277.0	E84
	Other	30.3, 30.4, 32.4, 32.41, 32.49, 32.5, 32.50, 32.59	0B110Z4, 0B113Z4, 0B114Z4, 0BTC0ZZ, 0BTC4ZZ, 0BTD0ZZ, 0BTD4ZZ, 0BTF0ZZ, 0BTF4ZZ, 0BTG0ZZ, 0BTG4ZZ, 0BTJ0ZZ, 0BTJ4ZZ, 0BTK0ZZ, 0BTK4ZZ, 0BTL0ZZ, 0BTL4ZZ, 0BTM0ZZ, 0BTM4ZZ, 0CTS0ZZ, 0CTS4ZZ, 0CTS7ZZ, 0CTS8ZZ
	Devices	519.0, V44.0, V55.0, V46.0, V46.1, 31.2, 31.21, 31.29, 31.41, 31.74, 33.21, 34.85, 96.55, 97.23	J95.00-J95.04, J95.09, Z43.0, Z93.0, Z99.0, J95.850, Z99.11, Z99.12, 0B110F4, 0B113F4, 0B114F4, 0B21XFZ, 0BHR0MZ, 0BHR3MZ, 0BHR4MZ, 0BHS0MZ, 0BHS3MZ, 0BHS4MZ, 0BW10FZ, 0BW13FZ, 0BW14FZ, 0JH604Z, 0JH634Z,

© 2026 O'Meara D et al. *JAMA Netw Open*.

Categories	Subcategories	ICD-9	ICD-10
			0JH804Z, 0JH834Z, 0WQ6XZ2, 3E1F78Z
	Transplantation	996.84,V42.6, 33.5, 33.50, 33.51, 33.52, 33.6	T86.810, T86.811, T86.819, Z94.2, 0BYC0Z0, 0BYC0Z1, 0BYC0Z2, 0BYD0Z0, 0BYD0Z1, 0BYD0Z2, 0BYF0Z0, 0BYF0Z1, 0BYF0Z2, 0BYG0Z0, 0BYG0Z1, 0BYG0Z2, 0BYH0Z0, 0BYH0Z1, 0BYH0Z2, 0BYJ0Z0, 0BYJ0Z1, 0BYJ0Z2, 0BYK0Z0, 0BYK0Z1, 0BYK0Z2, 0BYL0Z0, 0BYL0Z1, 0BYL0Z2, 0BYM0Z0, 0BYM0Z1, 0BYM0Z2
**Renal and Urologic**	Congenital anomalies	753.0-753.9	Q60-Q64
	Chronic renal failure	585	N18
	Other	V45.73, V45.74, 55.5, 55.51, 55.52, 55.53, 55.54, 56.4, 56.41, 56.42, 56.7, 56.71, 56.79, 57.7, 57.71, 57.79	Z90.5, Z90.6, 0T160Z8, 0T160ZA, 0T164Z8, 0T164ZA, 0T170Z8, 0T170ZA, 0T174Z8, 0T174ZA,

© 2026 O'Meara D et al. *JAMA Netw Open*.

Categories	Subcategories	ICD-9	ICD-10
			0T180Z8, 0T180ZA, 0T184Z8, 0T184ZA, 0TB60ZZ, 0TB63ZZ, 0TB64ZZ, 0TB67ZZ, 0TB68ZZ, 0TB70ZZ, 0TB73ZZ, 0TB74ZZ, 0TB77ZZ, 0TB78ZZ, 0TT00ZZ, 0TT04ZZ, 0TT10ZZ, 0TT14ZZ, 0TT20ZZ, 0TT24ZZ, 0TT60ZZ, 0TT64ZZ, 0TT67ZZ, 0TT68ZZ, 0TT70ZZ, 0TT74ZZ, 0TT77ZZ, 0TT78ZZ, 0TTB0ZZ, 0TTB4ZZ, 0TTB7ZZ, 0TTB8ZZ, 0TTD0ZZ, 0TTD4ZZ, 0TTD7ZZ, 0TTD8ZZ
	Chronic bladder diseases	344.61, 596.4, 596.53, 596.54	G83.4, N31.2, N31.9
	Devices	996.68, V44.5, V44.6, V45.1, V53.6, V55.5, V55.6, 38.95, 39.27, 39.42, 39.93, 39.94, 39.95, 54.98, 55.02, 55.03, 55.04, 55.12, 55.93, 55.94, 55.97, 56.5, 56.51, 56.52, 56.6, 56.61, 56.62, 56.72, 56.73, 56.74, 56.75, 57.2, 57.21, 57.22, 59.93, 59.94, 86.07, 96.45, 96.46, 96.47	T85.71XA, Z93.50-Z93.52, Z93.59, Z93.6, Z91.15, Z99.2, Z43.5, Z43.6, Z46.6, 031209D, 031209F, 03120AD, 03120AF, 03120JD, 03120JF, 03120KD, 03120KF, 03120ZD, 03120ZF, 031309D,

© 2026 O'Meara D et al. *JAMA Netw Open*.

Categories	Subcategories	ICD-9	ICD-10
			031309F, 03130AD, 03130AF, 03130JD, 03130JF, 03130KD, 03130KF, 03130ZD, 03130ZF, 031409D, 031409F, 03140AD, 03140AF, 03140JD, 03140JF, 03140KD, 03140KF, 03140ZD, 03140ZF, 031509D, 031509F, 03150AD, 03150AF, 03150JD, 03150JF, 03150KD, 03150KF, 03150ZD, 03150ZF, 031609D, 031609F, 03160AD, 03160AF, 03160JD, 03160JF, 03160KD, 03160KF, 03160ZD, 03160ZF, 031709D, 031709F, 03170AD, 03170AF, 03170JD, 03170JF, 03170KD, 03170KF, 03170ZD, 03170ZF, 031809D,

© 2026 O’Meara D et al. *JAMA Netw Open*.

Categories	Subcategories	ICD-9	ICD-10
			031809F, 03180AD, 03180AF, 03180JD, 03180JF, 03180KD, 03180KF, 03180ZD, 03180ZF, 031909F, 03190AF, 03190JF, 03190KF, 03190ZF, 031A09F, 031A0AF, 031A0JF, 031A0KF, 031A0ZF, 031B09F, 031B0AF, 031B0JF, 031B0KF, 031B0ZF, 031C09F, 031C0AF, 031C0JF, 031C0KF, 031C0ZF, 03WY0JZ, 03WY3JZ, 03WY4JZ, 03WYXJZ, 05HY33Z, 06HY33Z, 0JH60WZ, 0JH60XZ, 0JH63WZ, 0JH63XZ, 0JH80WZ, 0JH80XZ, 0JH83WZ, 0JH83XZ, 0JHD0WZ, 0JHD0XZ, 0JHD3WZ, 0JHD3XZ,

© 2026 O'Meara D et al. *JAMA Netw Open*.

Categories	Subcategories	ICD-9	ICD-10
			0JHF0WZ, 0JHF0XZ, 0JHF3WZ, 0JHF3XZ, 0JHL0WZ, 0JHL0XZ, 0JHL3WZ, 0JHL3XZ, 0JHM0WZ, 0JHM0XZ, 0JHM3WZ, 0JHM3XZ, 0T130ZB, 0T134ZB, 0T140ZB, 0T144ZB, 0T16079, 0T1607C, 0T1607D, 0T160J9, 0T160JC, 0T160JD, 0T160K9, 0T160KC, 0T160KD, 0T160Z9, 0T160ZC, 0T160ZD, 0T163JD, 0T16479, 0T1647C, 0T1647D, 0T164J9, 0T164JC, 0T164JD, 0T164K9, 0T164KC, 0T164KD, 0T164Z9, 0T164ZC, 0T164ZD, 0T17079, 0T1707C, 0T1707D, 0T170J9, 0T170JC, 0T170JD, 0T170K9,

© 2026 O'Meara D et al. *JAMA Netw Open*.

Categories	Subcategories	ICD-9	ICD-10
			0T170KC, 0T170KD, 0T170Z9, 0T170ZC, 0T170ZD, 0T173JD, 0T17479, 0T1747C, 0T1747D, 0T174J9, 0T174JC, 0T174JD, 0T174K9, 0T174KC, 0T174KD, 0T174Z9, 0T174ZC, 0T174ZD, 0T18079, 0T1807C, 0T1807D, 0T180J9, 0T180JC, 0T180JD, 0T180K9, 0T180KC, 0T180KD, 0T180Z9, 0T180ZC, 0T180ZD, 0T183JD, 0T18479, 0T1847C, 0T1847D, 0T184J9, 0T184JC, 0T184JD, 0T184K9, 0T184KC, 0T184KD, 0T184Z9, 0T184ZC, 0T184ZD, 0T1B0ZD, 0T1B4ZD, 0T25X0Z, 0T29X0Z, 0T29XYZ,

© 2026 O'Meara D et al. *JAMA Netw Open*.

Categories	Subcategories	ICD-9	ICD-10
			0T2BX0Z, 0T9000Z, 0T9030Z, 0T9040Z, 0T9070Z, 0T9080Z, 0T9100Z, 0T9130Z, 0T9140Z, 0T9170Z, 0T9180Z, 0T9370Z, 0T9380Z, 0T9470Z, 0T9480Z, 0TQ67ZZ, 0TQ77ZZ, 3E1K38Z, 3E1M39Z, 5A1D60Z
	Transplantation	996.81,V42.0, 55.6, 55.61, 55.69	T86.10-T86.12, Z94.0, 0TY00Z0, 0TY00Z1, 0TY00Z2, 0TY10Z0, 0TY10Z1, 0TY10Z2
**Gastrointestinal**	Congenital anomalies	750.3, 751.1-751.9	Q39.0-Q39.4, Q41-Q45
	Chronic liver disease and cirrhosis	571.4-571.9	K73, K74, K75.4, K760-K763, K765, K768
	Inflammatory bowel diseases	555.0-556.9	K50, K51
	Other	453.0, 557.1, 560.2, 564.7, V45.3, V45.72, V45.75, 25.3, 25.4, 42.42, 43.9, 43.91, 43.99, 45.63, 45.8, 45.81, 45.82, 45.83, 50.4, 52.6, 52.7, 54.71	I82.0, K55.1, K56.2, K59.3, Z98.0, Z90.3, Z90.49, 0CT70ZZ, 0CT7XZZ, 0D13079, 0D1307A, 0D1307B, 0D1607A, 0D160ZA,

© 2026 O’Meara D et al. *JAMA Netw Open*.

Categories	Subcategories	ICD-9	ICD-10
			0DT50ZZ, 0DT54ZZ, 0DT57ZZ, 0DT58ZZ, 0DT60ZZ, 0DT64ZZ, 0DT67ZZ, 0DT68ZZ, 0DT80ZZ, 0DT84ZZ, 0DT87ZZ, 0DT88ZZ, 0DT90ZZ, 0DT94ZZ, 0DT97ZZ, 0DT98ZZ, 0DTE0ZZ, 0DTE4ZZ, 0DTE7ZZ, 0DTE8ZZ, 0FT00ZZ, 0FT04ZZ, 0FTG0ZZ, 0FTG4ZZ
	Devices	536.4, V44.1-V44.4, V53.50, V53.51, V53.59, V55.1-V55.4, 42.1, 42.10, 42.11, 42.81, 43.1, 43.11, 43.19, 44.12, 44.3, 44.32, 44.38, 44.39, 46.1, 46.13, 46.2, 46.22, 46.23, 46.3, 46.32, 46.4, 46.40, 46.41, 46.43, 96.24, 96.36, 97.02	K94.20, K94.22, K94.23, K94.29, Z93.1-Z93.4, Z43.1-Z43.4, Z46.51, Z46.59, 0D11074, 0D110J4, 0D110K4, 0D110Z4, 0D113J4, 0D11474, 0D114J4, 0D114K4, 0D114Z4, 0D15074, 0D150J4, 0D150K4, 0D150Z4, 0D153J4, 0D15474, 0D154J4, 0D154K4, 0D154Z4,

© 2026 O'Meara D et al. *JAMA Netw Open*.

Categories	Subcategories	ICD-9	ICD-10
			0D16074,<br>0D160J4,<br>0D160J9,<br>0D160JA,<br>0D160K4,<br>0D160K9,<br>0D160KA,<br>0D160Z4,<br>0D163J4,<br>0D16474,<br>0D164J4,<br>0D164J9,<br>0D164JA,<br>0D164K4,<br>0D164K9,<br>0D164KA,<br>0D164Z4,<br>0D16874,<br>0D168J4,<br>0D168J9,<br>0D168JA,<br>0D168K4,<br>0D168K9,<br>0D168KA,<br>0D168Z4,<br>0D1B0Z4,<br>0D1B4Z4,<br>0D1B8Z4,<br>0D1H0Z4,<br>0D1H4Z4,<br>0D1H8Z4,<br>0D1K0Z4,<br>0D1K4Z4,<br>0D1K8Z4,<br>0D1L0Z4,<br>0D1L4Z4,<br>0D1L8Z4,<br>0D1N0Z4,<br>0D1N4Z4,<br>0D1N8Z4,<br>0D20X0Z,<br>0D20XUZ,<br>0D20XYZ,<br>0D787ZZ,<br>0D7E7ZZ,

© 2026 O'Meara D et al. *JAMA Netw Open*.

Categories	Subcategories	ICD-9	ICD-10
			0DBB7ZZ, 0DH50DZ, 0DH50UZ, 0DH53DZ, 0DH53UZ, 0DH54DZ, 0DH54UZ, 0DH57DZ, 0DH57UZ, 0DH58DZ, 0DH58UZ, 0DH63UZ, 0DH64UZ, 0DHA3UZ, 0DHA4UZ, 0DHA8UZ, 0DN87ZZ, 0DNE7ZZ, 0DW04UZ, 0DW08UZ, 0WQFXZ2, 3E1G78Z, 3E1H78Z
	Transplantation	996.82,996.86,996.87,V42.7, V42.83, V42.84, 46.97, 50.5, 50.51, 50.59, 52.8, 52.80, 52.82, 52.83, 52.84, 52.85, 52.86	T86.40-T86.42, T86.890, T86.891, T86.899, T86.850, T86.851, T86.859, Z94.4, Z94.82, Z94.83, 0DY80Z0, 0DY80Z1, 0DY80Z2, 0DYE0Z0, 0DYE0Z1, 0DYE0Z2, 0FY00Z0, 0FY00Z1, 0FY00Z2, 0FYG0Z0, 0FYG0Z1, 0FYG0Z2, 3E030U0, 3E030U1, 3E033U0, 3E033U1, 3E0J3U0,

© 2026 O’Meara D et al. *JAMA Netw Open*.

Categories	Subcategories	ICD-9	ICD-10
			3E0J3U1, 3E0J7U0, 3E0J7U1, 3E0J8U0, 3E0J8U1
Hematologic or immunologic	Hereditary anemias	282.0-282.6	D55-D58
	Aplastic anemias	284	D60-D61, D71
	Hereditary immunodeficiency	279.0-279.9, 288.1, 288.2, 446.1	D80-D89, D72.0, M30.3, M35.9
	Coagulation/hemorrhagic	286.0, 286.3, 287.32, 287.33, 287.39	D66, D68.2, D69.41, D69.42, D69.49
	Leukopenia	288.01, 288.02	D70.0, D70.4
	Hemophagocytic Syndromes	288.4	D76.1-D76.3
	Sarcoidosis	135	D86.9
	Acquired immunodeficiency	042-044	B20-B24
	Polyarteritis nodosa and related conditions	446.0, 446.21, 446.4-446.7	M30.0, M31.0, M31.1, M31.30, M31.4, M31.6
	Diffuse diseases of connective tissue	710.0, 710.1, 710.3	M32.10, M33.90, M34.0, M34.1, M34.9
	Other	41.5	07TP0ZZ, 07TP4ZZ
	Devices	N/A	N/A
	Transplantation	41.00, 41.01, 41.02, 41.03, 41.04, 41.05, 41.06, 41.07, 41.08, 41.09, 41.94	07YP0Z0, 07YP0Z1, 07YP0Z2, 30230AZ, 30230G0, 30230G1, 30230X0, 30230X1, 30230Y0, 30230Y1, 30233AZ, 30233G0, 30233G1, 30233X0, 30233X1, 30233Y0, 30233Y1,

© 2026 O'Meara D et al. *JAMA Netw Open*.

Categories	Subcategories	ICD-9	ICD-10
			30240AZ, 30240G0, 30240G1, 30240X0, 30240X1, 30240Y0, 30240Y1, 30243AZ, 30243G0, 30243G1, 30243X0, 30243X1, 30243Y0, 30243Y1, 30250G0, 30250G1, 30250X0, 30250X1, 30250Y0, 30250Y1, 30253G0, 30253G1, 30253X0, 30253X1, 30253Y0, 30253Y1, 30260G0, 30260G1, 30260X0, 30260X1, 30260Y0, 30260Y1, 30263G0, 30263G1, 30263X0, 30263X1, 30263Y0, 30263Y1
**Metabolic**	Amino acid metabolism	270.0-270.9	E70.0, E70.2, E70.3, E70.4, E70.8, E71.0-E71.5, E72.0-E72.4, E72.8, E72.9

© 2026 O'Meara D et al. *JAMA Netw Open*.

Categories	Subcategories	ICD-9	ICD-10
	Carbohydrate metabolism	271.0-271.9	E74.0-E74.4, E74.8, E74.9
	Lipid metabolism	272.0-272.9	E75, E77.0, E77.1, E78.0-E78.4, E78.5-E78.9, E88.1, E88.8
	Storage disorder	277.3, 277.5	E76.0-E76.3, E85
	Other metabolic disorders	275.0-275.3, 277.2,277.6, 277.8-277.9	277.4, E79.1, E79.8, E80.4-E80.7, E83.0, E83.1, E83.3, E83.4, D84.1, E88, H49.8
	Endocrine disorders	243, 253.2, 253.5, 253.6, 235.9, 255.0, 255.13, 255.2, 06.4, 06.52, 06.81, 07.3, 07.64, 07.65, 07.68, 07.69, 62.4, 62.41, 64.5, 65.5, 65.51, 65.53, 65.6, 65.61, 65.63, 68.4, 68.41, 68.49, 68.5, 68.51, 68.59, 68.6, 68.61, 68.69, 68.7, 68.71, 68.79	E00.9, E23.0, E23.2, E22.2, E23.3, E23.7, E24.0, E24.2, E24.3, E24.8, E24.9, E26.81, E25.0, E25.8, E25.9, 0GT00ZZ, 0GT04ZZ, 0GT40ZZ, 0GT44ZZ, 0GTK0ZZ, 0GTK4ZZ, 0GTR0ZZ, 0GTR4ZZ, 0UT20ZZ, 0UT24ZZ, 0UT27ZZ, 0UT28ZZ, 0UT2FZZ, 0UT40ZZ, 0UT44ZZ, 0UT47ZZ, 0UT48ZZ, 0UT70ZZ, 0UT74ZZ, 0UT90ZZ, 0UT94ZZ, 0UT97ZZ, 0UT98ZZ, 0UT9FZZ, 0UTC0ZZ,

© 2026 O'Meara D et al. *JAMA Netw Open*.

Categories	Subcategories	ICD-9	ICD-10
			0UTC7ZZ, 0UTC8ZZ, 0VTC0ZZ, 0VTC4ZZ, 0W4M070, 0W4M0J0, 0W4M0K0, 0W4M0Z0, 0W4N071, 0W4N0J1, 0W4N0K1, 0W4N0Z1
	Devices	V45.85, V53.91, 86.06	Z46.81, Z96.41, 0JH60VZ, 0JH63VZ, 0JH70VZ, 0JH73VZ, 0JH80VZ, 0JH83VZ, 0JHD0VZ, 0JHD3VZ, 0JHF0VZ, 0JHF3VZ, 0JHG0VZ, 0JHG3VZ, 0JHH0VZ, 0JHH3VZ, 0JHL0VZ, 0JHL3VZ, 0JHM0VZ, 0JHM3VZ, 0JHN0VZ, 0JHN3VZ, 0JHP0VZ, 0JHP3VZ, 0JHT0VZ, 0JHT3VZ
	Transplantation	N/A	N/A
**Other Congenital or Genetic Defect**	Chromosomal anomalies	758.0-758.9	Q90.9, Q91.3, Q91.4, Q91.7, Q92.8, Q93, Q95.0, Q96.9, Q97, Q98, Q99.8, Q99.9

© 2026 O'Meara D et al. *JAMA Netw Open*.

Categories	Subcategories	ICD-9	ICD-10
	Bone and join anomalies	259.4, 737.3, 756.0-756.5	E34.3, M41.0, M41.2, M41.30, M41.8, M41.9, M43.30, M96.5, Q72.2, Q75.0, Q75.2, Q75.9, Q76.0-Q76.2, Q76.4-Q76.7, Q77, Q78.0-Q78.4, Q78.8, Q78.9
	Diaphragm and abdominal wall	553.3, 756.6, 756.7	K44.9, Q79.0-Q79.5, Q79.9, Q79.59
	Other congenital anomalies	757.39, 759.7-759.9	Q81, Q87.1-Q87.3, Q87.40, Q87.81, Q87.89, Q89.7, Q89.9, Q99.2
**Malignancy**	Neoplasms	140-209, 230-239, 00.10, 99.25	C00-C96, D01-D09, D3A.0, D37-D49, Q85.0, 3E00X05, 3E01305, 3E02305, 3E03005, 3E03305, 3E04005, 3E04305, 3E05005, 3E05305, 3E06005, 3E06305, 3E0A305, 3E0F305, 3E0F705, 3E0F805, 3E0G305, 3E0G705, 3E0G805, 3E0H305, 3E0H705, 3E0H805, 3E0J305, 3E0J705,

© 2026 O’Meara D et al. *JAMA Netw Open*.

Categories	Subcategories	ICD-9	ICD-10
			3E0J805, 3E0K305, 3E0K705, 3E0K805, 3E0L305, 3E0L705, 3E0M305, 3E0M705, 3E0N305, 3E0N705, 3E0N805, 3E0P305, 3E0P705, 3E0P805, 3E0Q305, 3E0Q705, 3E0R305, 3E0S305, 3E0V305, 3E0W305, 3E0Y305, 3E0Y705
	Devices	N/A	N/A
	Transplantation	996.85,V42.81, V42.82	T86.00-T86.02, T86.09, Z94.81, Z94.84
**Premature and Neonatal**	Fetal malnutrition	764.01, 764.02, 764.11, 764.12, 764.21, 764.22, 764.91, 764.92	P05.01, P05.11, P05.02, P05.12, P05.2, P05.9
	Extreme immaturity	765.01, 765.02, 765.11, 765.12, 765.21-765.23	P07.01, P07.02, P07.21-P07.25
	Cerebral hemorrhage at birth	767.0	P10.0, P10.1, P10.4, P52.4, P52.8
	Spinal cord injury at birth	767.4	P11.5
	Birth asphyxia	768.5, 768.9	P21.0, P21.9, P84
	Respiratory diseases	770.2, 770.7	P25.0-P25.3, P25.8, P27.0, P27.1, P27.8
	Hypoxic-ischemic encephalopathy	768.7	P91.6
	Other	771.0, 771.1, 772.13, 772.14, 773.3, 773.4, 774.7, 776.5,	P35.0, P35.1, P25.21, P25.22,

© 2026 O'Meara D et al. *JAMA Netw Open*.

Categories	Subcategories	ICD-9	ICD-10
		777.53, 778.0, 779.7	P56.0, P57.0, P57.8, P61.3, P61.4, P77.3, P83.2, P91.2
**Miscellaneous, Not Elsewhere Classified**	Devices	996.4, 996.66, 996.67, 996.9, V46.2 81.00, 81.01, 81.02, 81.03, 81.04, 81.05, 81.06, 81.07, 81.08, 81.09, 81.30, 81.31, 81.32, 81.33, 81.34, 81.35, 81.36, 81.37, 81.38, 81.39, 84.51	T84.019A, T84.029A, T84.039A, T84.049A, T84.059A, T84.069A, T84.099A, T84.498A, T84.119A, T84.129A, T84.199A, T84.498A, T84.50XA, T84.60XA, , T84.7XXA, T86.90-T86.92, T86.99, T86.10-T86.12, T86.40-T86.42, T86.20-T86.22, T86.810, T86.811, T86.819, T86.00-T86.02, T86.09, T86.890, T86.891, T86.899, T86.850, T86.851, T86.859, T86.5, T86.890, T86.891, T86.899, T87.0X9, T87.1X9, T87.2, Y83.1, Y83.3, Z99.81, 0RG00J0, 0RG00J1, 0RG00JJ, 0RG00K0, 0RG00K1, 0RG00KJ, 0RG00Z0, 0RG00Z1, 0RG00ZJ, 0RG03J0,

© 2026 O'Meara D et al. *JAMA Netw Open*.

Categories	Subcategories	ICD-9	ICD-10
			0RG03J1, 0RG03JJ, 0RG03K0, 0RG03K1, 0RG03KJ, 0RG03Z0, 0RG03Z1, 0RG03ZJ, 0RG04J0, 0RG04J1, 0RG04JJ, 0RG04K0, 0RG04K1, 0RG04KJ, 0RG04Z0, 0RG04Z1, 0RG04ZJ, 0RG10J0, 0RG10J1, 0RG10JJ, 0RG10K0, 0RG10K1, 0RG10KJ, 0RG10Z0, 0RG10Z1, 0RG10ZJ, 0RG13J0, 0RG13J1, 0RG13JJ, 0RG13K0, 0RG13K1, 0RG13KJ, 0RG13Z0, 0RG13Z1, 0RG13ZJ, 0RG14J0, 0RG14J1, 0RG14JJ, 0RG14K0, 0RG14K1, 0RG14KJ, 0RG14Z0, 0RG14Z1, 0RG14ZJ, 0RG40J0, 0RG40J1, 0RG40JJ, 0RG40K0, 0RG40K1, 0RG40KJ, 0RG40Z0,

© 2026 O’Meara D et al. *JAMA Netw Open*.

Categories	Subcategories	ICD-9	ICD-10
			0RG40Z1, 0RG40ZJ, 0RG43J0, 0RG43J1, 0RG43JJ, 0RG43K0, 0RG43K1, 0RG43KJ, 0RG43Z0, 0RG43Z1, 0RG43ZJ, 0RG44J0, 0RG44J1, 0RG44JJ, 0RG44K0, 0RG44K1, 0RG44KJ, 0RG44Z0, 0RG44Z1, 0RG44ZJ, 0RG60J0, 0RG60J1, 0RG60JJ, 0RG60K0, 0RG60K1, 0RG60KJ, 0RG60Z0, 0RG60Z1, 0RG60ZJ, 0RG63J0, 0RG63J1, 0RG63JJ, 0RG63K0, 0RG63K1, 0RG63KJ, 0RG63Z0, 0RG63Z1, 0RG63ZJ, 0RG64J0, 0RG64J1, 0RG64JJ, 0RG64K0, 0RG64K1, 0RG64KJ, 0RG64Z0, 0RG64Z1, 0RG64ZJ, 0RGA0J0, 0RGA0J1, 0RGA0JJ,

© 2026 O’Meara D et al. *JAMA Netw Open*.

Categories	Subcategories	ICD-9	ICD-10
			0RGA0K0, 0RGA0K1, 0RGA0KJ, 0RGA0Z0, 0RGA0Z1, 0RGA0ZJ, 0RGA3J0, 0RGA3J1, 0RGA3JJ, 0RGA3K0, 0RGA3K1, 0RGA3KJ, 0RGA3Z0, 0RGA3Z1, 0RGA3ZJ, 0RGA4J0, 0RGA4J1, 0RGA4JJ, 0RGA4K0, 0RGA4K1, 0RGA4KJ, 0RGA4Z0, 0RGA4Z1, 0RGA4ZJ, 0SG00J0, 0SG00J1, 0SG00JJ, 0SG00K0, 0SG00K1, 0SG00KJ, 0SG00Z0, 0SG00Z1, 0SG00ZJ, 0SG03J0, 0SG03J1, 0SG03JJ, 0SG03K0, 0SG03K1, 0SG03KJ, 0SG03Z0, 0SG03Z1, 0SG03ZJ, 0SG04J0, 0SG04J1, 0SG04JJ, 0SG04K0, 0SG04K1, 0SG04KJ, 0SG04Z0, 0SG04Z1, 0SG04ZJ

© 2026 O’Meara D et al. *JAMA Netw Open*.

Categories	Subcategories	ICD-9	ICD-10
	Transplantation	996.80, 6.88, 996.89, 00.91, 00.92, 00.93	T86.5, T86.90-T86.92, T86.99, T86.890, T86.891, T86.899

© 2026 O'Meara D et al. *JAMA Netw Open*.

eTable 3. *CPT* and HCPCS Codes for Psychotherapy, Neurodevelopmental Testing, Early Intervention, Neuroimaging, Physical Therapy, Occupational Therapy, Speech and Language Therapy, Audiology, and Neurological Testing

ND CPT	Description	Category
90816	Psychotherapy, 20-30 min, Inpatient	Psychotherapy
90834	Psychotherapy, 20-30 min, Outpatient	Psychotherapy
90832	Psychotherapy, 20-30 min, Outpatient	Psychotherapy
90818	Psychotherapy, 45-50 min, Inpatient	Psychotherapy
90806	Psychotherapy, 45-50 min, Outpatient	Psychotherapy
90834	Psychotherapy, 45-50 min, Outpatient	Psychotherapy
90821	Psychotherapy, 60+ min, Inpatient	Psychotherapy
90808	Psychotherapy, 60+ min, Outpatient	Psychotherapy
90837	Psychotherapy, 60+ min, Outpatient	Psychotherapy
90845	Psychoanalysis	Psychotherapy
90846	Family Psychotherapy, wOut Pt	Psychotherapy
90847	Family Psychotherapy, with Pt	Psychotherapy
90849	Multiple-family Group Psychotherapy	Psychotherapy
90853	Group Psychotherapy	Psychotherapy
90839	Psychotherapy for Crisis	Psychotherapy
90785	Interactive Complexity (add on)	Psychotherapy
90862	Pharmacological Management (Add on to psychotherapy)	Psychotherapy
90863	Pharmacological Management (Add on to psychotherapy)	Psychotherapy
90801	Psychiatric Diagnostic Evaluation	Psychiatric Diagnostic Evaluation
90791	Psychiatric Diagnostic Evaluation	Psychiatric Diagnostic Evaluation
96116	Neurobehavioral Status Exam	Psych/Neuropsychological Testing
96121	Neurobehavioral Status Exam	Psych/Neuropsychological Testing
96101	Psychological testing by a QHP	Psych/Neuropsychological Testing
96130	Psychological testing evaluation services by a QHP	Psych/Neuropsychological Testing
96131	Psychological testing evaluation services by a QHP, +30	Psych/Neuropsychological Testing
96136	Psychological test administration by a QHP	Psych/Neuropsychological Testing
96137	Psychological test administration by a QHP, +30	Psych/Neuropsychological Testing

© 2026 O'Meara D et al. *JAMA Netw Open*.

96102	Psychological testing by a technician	Psych/Neuropsychological Testing
96138	Psychological test administration by a technician	Psych/Neuropsychological Testing
96139	Psychological test administration by a technician, +30	Psych/Neuropsychological Testing
96118	Neuropsychological Testing by a QHP	Psych/Neuropsychological Testing
96119	Neuropsychological Testing by a technician	Psych/Neuropsychological Testing
96132	Neuropsychological testing evaluation services by a QHP	Psych/Neuropsychological Testing
96133	Neuropsychological testing evaluation services by a QHP, +30	Psych/Neuropsychological Testing
96136	Neuropsychological test administration by a QHP	Psych/Neuropsychological Testing
96137	Neuropsychological test administration by a QHP, +30	Psych/Neuropsychological Testing
96138	Neuropsychological Testing by a technician	Psych/Neuropsychological Testing
96139	Neuropsychological Testing by a technician, +30	Psych/Neuropsychological Testing
96150	H&B Assessment, Initial	Other EI services
96151	H&B Assessment, Reassessment	Other EI services
96156	H&B Assessment, Reassessment	Other EI services
96152	H&B Intervention	Other EI services
96158	H&B Intervention	Other EI services
96159	H&B Intervention	Other EI services
96153	H&B Intervention, Group	Other EI services
96164	H&B Intervention, Group	Other EI services
96165	H&B Intervention, Group	Other EI services
96154	H&B Intervention, Family with Pt	Other EI services
96167	H&B Intervention, Family with Pt	Other EI services
96168	H&B Intervention, Family with Pt	Other EI services
96155	H&B Intervention, Family wOut Pt	Other EI services
96170	H&B Intervention, Family wOut Pt	Other EI services
96171	H&B Intervention, Family wOut Pt	Other EI services
H2014	Skills training and development, per 15 minutes	Other EI services
T1023	Screening to determine the appropriateness of consideration of an individual for participation in a specified program, project or treatment protocol, per encounter	Other EI services
T1027	Family training and counseling for child development, per 15 minutes	Other EI services

© 2026 O'Meara D et al. *JAMA Netw Open*.

T1028	Assessment of home, physical and family environment, to determine suitability to meet patient's medical needs	Other EI services
T2024	Service assessment/plan of care development, waiver	Other EI services
96110	Developmental Screening	Developmental/Behavioral Screening and Testing
96112	Developmental Test Administration by QHP	Developmental/Behavioral Screening and Testing
96113	Developmental Test Administration by QHP, +30	Developmental/Behavioral Screening and Testing
96127	Brief emotional/behavioral assessment	Developmental/Behavioral Screening and Testing
**Neuro Imaging CPT**	**Description**	**Category**
70450	Ct head/brain w/o dye	CT
70460	Ct head/brain w/dye	CT
70470	Ct head/brain w/o & w/dye	CT
70480	Ct orbit/ear/fossa w/o dye	CT
70481	Ct orbit/ear/fossa w/dye	CT
70482	Ct orbit/ear/fossa w/o&w/dye	CT
70486	Ct maxillofacial w/o dye	CT
70487	Ct maxillofacial w/dye	CT
70488	Ct maxillofacial w/o & w/dye	CT
70490	Ct soft tissue neck w/o dye	CT
70491	Ct soft tissue neck w/dye	CT
70492	Ct sft tsue nck w/o & w/dye	CT
72125	Ct neck spine w/o dye	CT
72126	Ct neck spine w/dye	CT
72127	Ct neck spine w/o & w/dye	CT
70336	Magnetic image jaw joint	MRI
70540	Mri orbit/face/neck w/o dye	MRI
70542	Mri orbit/face/neck w/dye	MRI
70543	Mri orbt/fac/nck w/o &w/dye	MRI
70551	Mri brain stem w/o dye	MRI
70552	Mri brain stem w/dye	MRI
70553	Mri brain stem w/o & w/dye	MRI
70557	Mri brain w/o dye	MRI

© 2026 O'Meara D et al. *JAMA Netw Open*.

70558	Mri brain w/dye	MRI
70559	Mri brain w/o & w/dye	MRI
72141	Mri neck spine w/o dye	MRI
72142	Mri neck spine w/dye	MRI
72156	Mri neck spine w/o & w/dye	MRI
76536	Us exam of head and neck	US
**PT/OT CPT**	**Description**	**Category**
29065	Application of long arm cast	PT/OT
29075	Application of forearm cast	PT/OT
29085	Apply hand/wrist cast	PT/OT
29086	Apply finger cast	PT/OT
29105	Apply long arm splint	PT/OT
29125	Apply forearm splint	PT/OT
29126	Apply forearm splint	PT/OT
29130	Application of finger splint	PT/OT
29131	Application of finger splint	PT/OT
29200	Strapping of chest	PT/OT
29240	Strapping of shoulder	PT/OT
29260	Strapping of elbow or wrist	PT/OT
29280	Strapping of hand or finger	PT/OT
29345	Application of long leg cast	PT/OT
29355	Application of long leg cast	PT/OT
29365	Application of long leg cast	PT/OT
29405	Apply short leg cast	PT/OT
29425	Apply short leg cast	PT/OT
29445	Apply rigid leg cast	PT/OT
29505	Application long leg splint	PT/OT
29515	Application lower leg splint	PT/OT
29520	Strapping of hip	PT/OT
29530	Strapping of knee	PT/OT
29540	Strapping of ankle and/or ft	PT/OT
29550	Strapping of toes	PT/OT
29580	Application of paste boot	PT/OT
29799	Casting/strapping procedure	PT/OT
90901	Biofeedback train any meth	PT/OT
90912	Bfb training 1st 15 min	PT/OT
90913	Bfb training ea addl 15 min	PT/OT

© 2026 O’Meara D et al. *JAMA Netw Open*.

95851	Range of motion measurements	PT/OT
95852	Range of motion measurements	PT/OT
96125	Cognitive test by hc pro	PT/OT
97010	Hot or cold packs therapy	PT/OT
97012	Mechanical traction therapy	PT/OT
97016	Vasopneumatic device therapy	PT/OT
97018	Paraffin bath therapy	PT/OT
97022	Whirlpool therapy	PT/OT
97024	Diathermy eg microwave	PT/OT
97026	Infrared therapy	PT/OT
97028	Ultraviolet therapy	PT/OT
97032	Electrical stimulation	PT/OT
97033	Electric current therapy	PT/OT
97034	Contrast bath therapy	PT/OT
97035	Ultrasound therapy	PT/OT
97036	Hydrotherapy	PT/OT
97039	Physical therapy treatment	PT/OT
97110	Therapeutic exercises	PT/OT
97112	Neuromuscular reeducation	PT/OT
97113	Aquatic therapy/exercises	PT/OT
97116	Gait training therapy	PT/OT
97124	Massage therapy	PT/OT
97129	Ther ivntj 1st 15 min	PT/OT
97130	Ther ivntj ea addl 15 min	PT/OT
97139	Physical medicine procedure	PT/OT
97140	Manual therapy 1/> regions	PT/OT
97150	Group therapeutic procedures	PT/OT
97161	Pt eval low complex 20 min	PT/OT
97162	Pt eval mod complex 30 min	PT/OT
97163	Pt eval high complex 45 min	PT/OT
97164	Pt re-eval est plan care	PT/OT
97165	Ot eval low complex 30 min	PT/OT
97166	Ot eval mod complex 45 min	PT/OT
97167	Ot eval high complex 60 min	PT/OT
97168	Ot re-eval est plan care	PT/OT
97530	Therapeutic activities	PT/OT
97533	Sensory integration	PT/OT
97535	Self care mngment training	PT/OT
97537	Community/work reintegration	PT/OT

© 2026 O'Meara D et al. *JAMA Netw Open*.

97542	Wheelchair mngment training	PT/OT
97597	Rmvl devital tis 20 cm/<	PT/OT
97598	Rmvl devital tis addl 20cm/<	PT/OT
97602	Wound(s) care non-selective	PT/OT
97605	Neg press wound tx <=50 cm	PT/OT
97606	Neg press wound tx >50 cm	PT/OT
97607	Neg press wnd tx <=50 sq cm	PT/OT
97608	Neg press wound tx >50 cm	PT/OT
97750	Physical performance test	PT/OT
97755	Assistive technology assess	PT/OT
97760	Orthotic mgmt&traing 1st enc	PT/OT
97761	Prosthetic traing 1st enc	PT/OT
97763	Orthc/prostc mgmt sbsq enc	PT/OT
97799	Physical medicine procedure	PT/OT
G0281	Application of long arm cast	PT/OT
G0283	Application of forearm cast	PT/OT
G0329	Apply hand/wrist cast	PT/OT
98966	Hc pro phone call 5-10 min	PT/OT Telehealth
98967	Hc pro phone call 11-20 min	PT/OT Telehealth
98968	Hc pro phone call 21-30 min	PT/OT Telehealth
98970	Qnhp ol dig assmt&mgmt 5-10	PT/OT Telehealth
98971	Qnhp ol dig assmt&mgmt 11-20	PT/OT Telehealth
98972	Qnhp ol dig assmt&mgmt 21+	PT/OT Telehealth
98975	Rem ther mntr 1st setup&edu	PT/OT Telehealth
98976	Rem ther mntr dev sply resp	PT/OT Telehealth
98977	Rem ther mntr dv sply mscskl	PT/OT Telehealth
98980	Rem ther mntr 1st 20 min	PT/OT Telehealth
98981	Rem ther mntr ea addl 20 min	PT/OT Telehealth
G2010	Remot image submit by pt	PT/OT Telehealth
G2012	Brief check in by md/qhp	PT/OT Telehealth
G2250	Remot img sub by pt, non e/m	PT/OT Telehealth
G2251	Brief chkin, 5-10, non-e/m	PT/OT Telehealth
**SLP CPT**	**Description**	**Category**
31579	Diagnostic laryngoscopy with stroboscopy	SLP-specific
92507	Treatment of speech, language, voice, communication, and/or auditory processing disorder; **individual**	SLP-specific

© 2026 O'Meara D et al. *JAMA Netw Open*.

92508	Treatment of speech, language, voice, communication, and/or auditory processing disorder; **group, two or more individuals**	SLP-specific
92511	Nasopharyngoscopy with endoscope (separate procedure)	SLP-specific
92520	Laryngeal function studies (i.e., aerodynamic testing and acoustic testing)	SLP-specific
92521	Evaluation of speech fluency (eg, stuttering, cluttering)	SLP-specific
92522	Evaluation of speech sound production (eg, articulation, phonological process, apraxia, dysarthria);	SLP-specific
92523	Evaluation of speech sound production (eg, articulation, phonological process, apraxia, dysarthria); **with** evaluation of language comprehension and expression (eg, receptive and expressive language)	SLP-specific
92524	Behavioral and qualitative analysis of voice and resonance	SLP-specific
92526	Treatment of swallowing dysfunction and/or oral function for feeding	SLP-specific
92597	Evaluation for use and/or fitting of voice prosthetic device to supplement oral speech	SLP-specific
92605	Evaluation for prescription for non-speech generating AAC device, face-to-face with the patient; **first hour**	SLP-specific
92618	Evaluation for prescription for non-speech generating AAC device, face-to-face with the patient; **each additional 30 minutes**	SLP-specific
92606	Therapeutic services for use of non-speech generating devices, including programming and modification	SLP-specific
92607	Evaluation for prescription of speech-generating AAC device; **first hour**	SLP-specific
92608	Evaluation for prescription of speech-generating AAC device; e**ach additional 30 minutes**	SLP-specific
92609	Therapeutic services for use of speech-generating device, including programming and modification	SLP-specific
92610	Evaluation of oral and pharyngeal swallowing function	SLP-specific
92611	Motion fluoroscopic evaluation of swallowing function by cine or video recording	SLP-specific
92612	Flexible fiberoptic endoscopic evaluation of swallowing by cine or video recording (FEES);	SLP-specific
92613	Flexible fiberoptic endoscopic evaluation of swallowing by cine or video recording (FEES); **interpretation and report only**	SLP-specific
92614	Flexible fiberoptic endoscopic evaluation, laryngeal sensory testing by cine or video recording;	SLP-specific
92615	Flexible fiberoptic endoscopic evaluation, laryngeal sensory testing by cine or video recording; **interpretation and report only**	SLP-specific

© 2026 O’Meara D et al. *JAMA Netw Open*.

92616	Flexible fiberoptic endoscopic evaluation of swallowing and laryngeal sensory testing by cine or video recording (FEESST);	SLP-specific
92617	Flexible fiberoptic endoscopic evaluation of swallowing and laryngeal sensory testing by cine or video recording (FEESST); **interpretation and report only**	SLP-specific
92626	Evaluation of auditory function for surgically implanted device(s) candidacy or postoperative status of a surgically implanted device(s); **first hour**	SLP-specific
92627	Evaluation of auditory function for surgically implanted device(s) candidacy or postoperative status of a surgically implanted device(s); **each additional 15 minutes** (list separately in addition to code for primary procedure)	SLP-specific
92630	Auditory rehabilitation; **pre-lingual hearing loss**	SLP-specific
92633	Auditory rehabilitation; **post-lingual hearing loss**	SLP-specific
96105	Assessment of aphasia (includes assessment of expressive and receptive speech and language function, language comprehension, speech production ability, reading, spelling, writing, eg, by Boston Diagnostic Aphasia Examination) with interpretation and report, per hour	SLP-specific
96112	Developmental test administration (including assessment of fine and/or gross motor, language, cognitive level, social, memory and/or executive functions by standardized developmental instruments when performed), by physician or other qualified health care professional, with interpretation and report; **first hour**	SLP-specific
96113	Developmental test administration (including assessment of fine and/or gross motor, language, cognitive level, social, memory and/or executive functions by standardized developmental instruments when performed), by physician or other qualified health care professional, with interpretation and report; **each additional 30 minutes** (List separately in addition to code for primary procedure.)	SLP-specific
96125	Standardized cognitive performance testing (eg, Ross Information Processing Assessment) per hour of a qualified health care professional's time, both face-to-face time administering tests to the patient and time interpreting these test results and preparing the report.	SLP-specific
97129	Therapeutic interventions that focus on cognitive function (eg, attention, memory, reasoning, executive function, problem solving, and/or pragmatic functioning) and compensatory strategies to manage the performance of an activity (eg, managing time or schedules, initiating, organizing and sequencing tasks), direct (one-on-one) patient contact; **initial 15 minutes**	SLP-specific

© 2026 O’Meara D et al. *JAMA Netw Open*.

97130	Therapeutic interventions that focus on cognitive function (eg, attention, memory, reasoning, executive function, problem solving, and/or pragmatic functioning) and compensatory strategies to manage the performance of an activity (eg, managing time or schedules, initiating, organizing and sequencing tasks), direct (one-on-one) patient contact; **each additional 15 minutes** (list separately in addition to code for primary procedure)	SLP-specific
97533	Sensory integrative techniques to enhance sensory processing and promote adaptive responses to environmental demands, direct (one-on-one) patient contact by the provider, each 15 minutes	SLP-specific
98970	Qualified nonphysician health care professional online digital assessment and management, for an established patient, for up to 7 days, cumulative time during the 7 days; **5-10 minutes**	SLP-specific
98971	Qualified nonphysician health care professional online digital assessment and management, for an established patient, for up to 7 days, cumulative time during the 7 days; **11-20 minutes**	SLP-specific
98972	Qualified nonphysician health care professional online digital assessment and management, for an established patient, for up to 7 days, cumulative time during the 7 days; **21 or more minutes**	SLP-specific
98975	Remote therapeutic monitoring (e.g., respiratory system status, musculoskeletal system status, therapy adherence, therapy response); **initial set-up and patient education on use of equipment**	SLP-specific
98976	Remote therapeutic monitoring (e.g., respiratory system status, musculoskeletal system status, therapy adherence, therapy response); **device(s) supply with scheduled (eg, daily) recording(s) and/or programmed alert(s) transmission to monitor respiratory system, each 30 days**	SLP-specific
98977	Remote therapeutic monitoring (e.g., respiratory system status, musculoskeletal system status, therapy adherence, therapy response); **device(s) supply with scheduled (eg, daily) recording(s) and/or programmed alert(s) transmission to monitor musculoskeletal system, each 30 days**	SLP-specific
98980	Remote therapeutic monitoring treatment management services, physician/other qualified health care professional time in a calendar month requiring at least one interactive communication with the patient/caregiver during the calendar month; **first 20 minutes**	SLP-specific
98981	Remote therapeutic monitoring treatment management services, physician/other qualified health care professional time in a calendar month requiring at least one interactive	SLP-specific

© 2026 O'Meara D et al. *JAMA Netw Open*.

	communication with the patient/caregiver during the calendar month; **each additional 20 minutes** (listed separately in addition to code for primary procedure)	
G0451	Developmental testing, with interpretation and report, per standardized instrument form	SLP-specific
G2250	Remote assessment of recorded video and/or images submitted by an established patient (e.g., store and forward), including interpretation with follow-up with the patient within 24 business hours, not originating from a related service provided within the previous 7 days nor leading to a service or procedure within the next 24 hours or soonest available appointment	SLP-specific
G2251	Brief communication technology-based service, e.g. virtual check-in, by a qualified health care professional who cannot report evaluation and management services, provided to an established patient, not originating from a related e/m service provided within the previous 7 days nor leading to a service or procedure within the next 24 hours or soonest available appointment; 5-10 minutes of medical discussion	SLP-specific
92700	Unlisted otorhinolaryngological service or procedure	SLP-specific
70371	Pharyngeal and speech evaluation, by cine or video	Other
74230	Swallowing function, with cineradiography/videoradiography	Other
90901	Biofeedback training	Other
96110	Developmental screening, with interpretation and report, per standardized instrument form	Other
97032	Electrical stimulation, manual, each 15 minutes	Other
97110	Therapeutic procedure, one or more areas, each 15 minutes; therapeutic exercises to develop strength and endurance, range of motion and flexibility	Other
97112	Neuromuscular reeducation of movement, balance, coordination, kinesthetic sense, posture, and/or proprioception for sitting and/or standing activities, each 15 minutes	Other
97150	Therapeutic procedure(s), group (2 or more individuals)	Other
97530	Therapeutic activities, direct (one-on-one) patient contact by the provider (use of dynamic activities to improve functional performance), each 15 minutes	Other
97535	Self-care/home management training (eg, activities of daily living and compensatory training, meal preparation, safety procedures, and instructions in use of assistive technology devices/adaptive equipment) direct one-on-one contact by provider, each 15 minutes	Other
97537	Community/work reintegration training (eg, shopping, transportation, money management, avocational activities	Other

© 2026 O'Meara D et al. *JAMA Netw Open*.

	and/or work environment/modification analysis, work task analysis, use of assistive technology devices/adaptive equipment) direct one-on-one contact by provider, each 15 minutes	
98966	Telephone assessment and management service provided by a qualified nonphysician health care professional to an established patient, parent, or guardian not originating from a related assessment and management service provided within the previous seven days nor leading to an assessment and management service or procedure with the next 24 hours or soonest available appointment; **5-10 minutes of medical discussion**	Other
98967	Telephone assessment and management service provided by a qualified nonphysician health care professional to an established patient, parent, or guardian not originating from a related assessment and management service provided within the previous seven days nor leading to an assessment and management service or procedure with the next 24 hours or soonest available appointment; **11-20 minutes of medical discussion**	Other
98968	Telephone assessment and management service provided by a qualified nonphysician health care professional to an established patient, parent, or guardian not originating from a related assessment and management service provided within the previous seven days nor leading to an assessment and management service or procedure with the next 24 hours or soonest available appointment; **21-30 minutes of medical discussion**	Other
G2252	Brief communication technology-based service, e.g. virtual check-in, by a physician or other qualified health care professional who can report evaluation and management services, provided to an established patient, not originating from a related e/m service provided within the previous 7 days nor leading to an e/m service or procedure within the next 24 hours or soonest available appointment; 11-20 minutes of medical discussion	Other
**Audio-logy CPT**	**Description**	**Category**
92517	Vestibular evoked myogenic potential testing, with interpretation and report; **cervical (cVEMP)**	Audiology-specific
92518	Vestibular evoked myogenic potential testing, with interpretation and report; **ocular (oVEMP)**	Audiology-specific
92519	Vestibular evoked myogenic potential testing, with interpretation and report; **cervical (cVEMP) and ocular (oVEMP)**	Audiology-specific

© 2026 O'Meara D et al. *JAMA Netw Open*.

92537	Caloric vestibular test with recording, bilateral; **bithermal** (i.e., one warm and one cool irrigation in each ear for a total of four irrigations)	Audiology-specific
92538	Caloric vestibular test with recording, bilateral; **monothermal** (i.e., one irrigation in each ear for a total of two irrigations)	Audiology-specific
92540	Basic vestibular evaluation, includes spontaneous nystagmus test with eccentric gaze fixation nystagmus, with recording, positional nystagmus test, minimum of 4 positions, with recording, optokinetic nystagmus test, bidirectional foveal and peripheral stimulation, with recording, and oscillating tracking test, with recording. (Do not report in conjunction with 92541, 92542, 92544, or 92545)	Audiology-specific
92541	Spontaneous nystagmus test, including gaze and fixation nystagmus, with recording. (Do not report 92541 in conjunction with 92540 or the set of 92542, 92544, and 92545)	Audiology-specific
92542	Positional nystagmus test, minimum of 4 positions, with recording. (Do not report 92542 in conjunction with 92540 or the set of 92541, 92544, and 92545)	Audiology-specific
92544	Optokinetic nystagmus test, bi-directional, foveal or peripheral stimulation, with recording (Do not report 92544 in conjunction with 92540 or the set of 92541, 92542, and 92545)	Audiology-specific
92545	Oscillating tracking test, with recording (Do not report 92545 in conjunction with 92540 or the set of 92541, 92542, and 92544)	Audiology-specific
92546	Sinusoidal vertical axis rotational testing	Audiology-specific
92547	Use of vertical electrodes (List separately in addition to code for primary procedure)	Audiology-specific
92548	Computerized dynamic posturography sensory organization test (CDP-SOT), **6 conditions (ie, eyes open, eyes closed, visual sway, platform sway, eyes closed platform sway, platform and visual sway)**, including interpretation and report;	Audiology-specific
92549	Computerized dynamic posturography sensory organization test (CDP-SOT), 6 conditions (ie, eyes open, eyes closed, visual sway, platform sway, eyes closed platform sway, platform and visual sway), including interpretation and report; **with motor control test (MCT) and adaptation test (ADT)**	Audiology-specific
92550	Tympanometry and reflex threshold measurements	Audiology-specific
92552	Pure tone audiometry (threshold); **air only**	Audiology-specific
92553	Pure tone audiometry (threshold); **air and bone**	Audiology-specific
92555	Speech audiometry threshold;	Audiology-specific

© 2026 O'Meara D et al. *JAMA Netw Open*.

92556	Speech audiometry threshold; **with speech recognition**	Audiology-specific
92557	Comprehensive audiometry threshold evaluation and speech recognition	Audiology-specific
92562	Loudness balance test, alternate binaural or monaural	Audiology-specific
92563	Tone decay test	Audiology-specific
92565	Stenger test, pure tone	Audiology-specific
92567	Tympanometry (impedance testing)	Audiology-specific
92568	Acoustic reflex testing; threshold	Audiology-specific
92570	Acoustic immittance testing, includes tympanometry (impedance testing), acoustic reflex threshold testing, and acoustic reflex decay testing	Audiology-specific
92571	Filtered speech test	Audiology-specific
92572	Staggered spondaic word test	Audiology-specific
92575	Sensorineural acuity level test	Audiology-specific
92576	Synthetic sentence identification test	Audiology-specific
92577	Stenger test, speech	Audiology-specific
92579	Visual reinforcement audiometry (VRA)	Audiology-specific
92582	Conditioning play audiometry	Audiology-specific
92583	Select picture audiometry	Audiology-specific
92584	Electrocochleography	Audiology-specific
92587	Distortion product evoked otoacoustic emissions; **limited evaluation** (to confirm the presence or absence of hearing disorder, 3-6 frequencies) or transient evoked otoacoustic emissions, with interpretation and report	Audiology-specific
92588	Distortion product evoked otoacoustic emissions; **comprehensive diagnostic evaluation** (quantitative analysis of outer hair cell function by cochlear mapping, minimum of 12 frequencies), with interpretation and report	Audiology-specific
92596	Ear protector attenuation measurements	Audiology-specific
92601	Diagnostic analysis of cochlear implant, patient under 7 years of age; **with programming**	Audiology-specific
92602	Diagnostic analysis of cochlear implant, patient under 7 years of age; **subsequent reprogramming**	Audiology-specific
92603	Diagnostic analysis of cochlear implant, age 7 years or older; **with programming**	Audiology-specific
92604	Diagnostic analysis of cochlear implant, age 7 years or older; **subsequent reprogramming**	Audiology-specific
92620	Evaluation of central auditory processing, with report; **initial 60 minutes**	Audiology-specific
92621	Evaluation of central auditory processing, with report; **each additional 15 minutes**	Audiology-specific
92625	Tinnitus assessment (includes pitch, loudness, matching, and masking)	Audiology-specific

© 2026 O’Meara D et al. *JAMA Netw Open*.

92626	Evaluation of auditory function for surgically implanted device(s) candidacy or postoperative status of a surgically implanted device(s); **first hour**	Audiology-specific
92627	Evaluation of auditory function for surgically implanted device(s) candidacy or postoperative status of a surgically implanted device(s); **each additional 15 minutes** (list separately in addition to code for primary procedure)	Audiology-specific
92640	Diagnostic analysis with programming of auditory brainstem implant, per hour	Audiology-specific
92651	Auditory evoked potentials; **for hearing status determination, broadband stimuli**, with interpretation and report	Audiology-specific
92652	Auditory evoked potentials; **for threshold estimation at multiple frequencies**, with interpretation and report	Audiology-specific
92653	Auditory evoked potentials; **neurodiagnostic**, with interpretation and report	Audiology-specific
92700	Unlisted otorhinolaryngological service or procedure	Audiology-specific
69209	Removal impacted cerumen using irrigation/lavage, unilateral	Other
69210	Removal impacted cerumen requiring instrumentation, unilateral (for bilateral procedure, report 69210)	Other
92507	Treatment of speech, language, voice, communication, and/or auditory processing disorder; individual	Other
92516	Facial nerve function studies (eg, electroneuronography)	Other
92551	Screening test, pure tone, air only	Other
92558	Evoked otoacoustic emissions, screening (qualitative measurement of distortion product or transient evoked otoacoustic emissions), automated analysis	Other
92590	Hearing aid examination and selection; **monaural**	Other
92591	Hearing aid examination and selection; **binaural**	Other
92592	Hearing aid check; **monaural**	Other
92593	Hearing aid check; **binaural**	Other
92594	Electroacoustic evaluation for hearing aid; **monaural**	Other
92595	Electroacoustic evaluation for hearing aid; **binaural**	Other
92630	Auditory rehabilitation; **pre-lingual hearing loss**	Other
92633	Auditory rehabilitation; **post-lingual hearing loss**	Other
92650	Auditory evoked potentials; screening of auditory potential with broadband stimuli, automated analysis	Other
95907	Nerve conduction studies; 1-2 studies	Other
95908	Nerve conduction studies; 3–4 studies	Other
95909	Nerve conduction studies; 5–6 studies	Other
95910	Nerve conduction studies; 7–8 studies	Other
95911	Nerve conduction studies; 9–10 studies	Other

© 2026 O’Meara D et al. *JAMA Netw Open*.

95912	Nerve conduction studies;11–12 studies	Other
95913	Nerve conduction studies; 13 or more studies	Other
95925	Somatosensory testing; in upper limbs	Other
95926	Somatosensory testing; in lower limbs	Other
95927	Somatosensory testing; in trunk or head	Other
95930	Visual evoked potential (VEP) testing central nervous system, checkerboard or flash	Other
95937	Neuromuscular junction testing (repetitive stimulation, paired stimuli), each nerve, any one method	Other
95940	Continuous intraoperative neurophysiology monitoring in the operating room, one on one monitoring requiring personal attendance, each 15 minutes (List separately in addition to code for primary procedure)	Other
95941	Continuous neurophysiology monitoring, from outside the operating room (remote or nearby) or for monitoring of more than one case while in the operating room, per hour (List separately in addition to code for primary procedure)	Other
98966	Telephone assessment and management service provided by a qualified nonphysician health care professional to an established patient, parent, or guardian not originating from a related assessment and management service provided within the previous seven days nor leading to an assessment and management service or procedure with the next 24 hours or soonest available appointment; **5-10 minutes of medical discussion**	Other
98967	Telephone assessment and management service provided by a qualified nonphysician health care professional to an established patient, parent, or guardian not originating from a related assessment and management service provided within the previous seven days nor leading to an assessment and management service or procedure with the next 24 hours or soonest available appointment; **11-20 minutes of medical discussion**	Other
98968	Telephone assessment and management service provided by a qualified nonphysician health care professional to an established patient, parent, or guardian not originating from a related assessment and management service provided within the previous seven days nor leading to an assessment and management service or procedure with the next 24 hours or soonest available appointment; **21-30 minutes of medical discussion**	Other
98970	Qualified nonphysician health care professional online digital assessment and management, for an established patient, for up to 7 days, cumulative time during the 7 days; **5-10 minutes**	Other

© 2026 O'Meara D et al. *JAMA Netw Open*.

98971	Qualified nonphysician health care professional online digital assessment and management, for an established patient, for up to 7 days, cumulative time during the 7 days; **11-20 minutes**	Other
98972	Qualified nonphysician health care professional online digital assessment and management, for an established patient, for up to 7 days, cumulative time during the 7 days; **21 or more minutes**	Other
98975	Remote therapeutic monitoring (e.g., respiratory system status, musculoskeletal system status, therapy adherence, therapy response); initial set-up and patient education on use of equipment	Other
98980	Remote therapeutic monitoring treatment management services, physician/other qualified health care professional time in a calendar month requiring at least one interactive communication with the patient/caregiver during the calendar month; **first 20 minutes**	Other
98981	Remote therapeutic monitoring treatment management services, physician/other qualified health care professional time in a calendar month requiring at least one interactive communication with the patient/caregiver during the calendar month; **each additional 20 minutes** (listed separately in addition to code for primary procedure)	Other
G0453	Continuous intraoperative neurophysiology monitoring, from outside the operating room (remote or nearby), per patient, (attention directed exclusively to one patient) each 15 minutes (list in addition to primary procedure)	Other
G2250	Remote assessment of recorded video and/or images submitted by an established patient (e.g., store and forward), including interpretation with follow-up with the patient within 24 business hours, not originating from a related service provided within the previous 7 days nor leading to a service or procedure within the next 24 hours or soonest available appointment	Other
G2251	Brief communication technology-based service, e.g. virtual check-in, by a qualified health care professional who cannot report evaluation and management services, provided to an established patient, not originating from a related e/m service provided within the previous 7 days nor leading to a service or procedure within the next 24 hours or soonest available appointment; 5-10 minutes of medical discussion	Other
**EI CPT**	**Description**	**Category**
96156	RVU 2.67 Health and behavior assessment	Health and behavior

© 2026 O'Meara D et al. *JAMA Netw Open*.

96158	RVU 1.82 Heath and Behavior Intervention	Health and behavior
96167	RVU 1.94 Heath and Behavior Intervention with family	Health and behavior
H2014	Skills training and development, per 15 minutes	EI-specific
T1023	Screening to determine the appropriateness of consideration of an individual for participation in a specified program, project or treatment protocol, per encounter	EI-specific
T1027	Family training and counseling for child development, per 15 minutes	EI-specific
T1028	Assessment of home, physical and family environment, to determine suitability to meet patient's medical needs	EI-specific
T2024	Service assessment/plan of care development, waiver	EI-specific
**Neuro CPT**	**Description**	**Category**
99202	Office o/p new sf 15-29 min	Office visit
99203	Office o/p new low 30-44 min	Office visit
99204	Office o/p new mod 45-59 min	Office visit
99205	Office o/p new high 60-74 min	Office visit
		Office visit
99211	Off/op est may x req phy/qhp	Office visit
99212	Office o/p est sf 10-19 min	Office visit
99213	Office o/p est low 20-29 min	Office visit
99214	Office o/p est mod 30-39 min	Office visit
99215	Office o/p est high 40-54 min	Office visit
		Office visit
99221	Initial hospital care sf/low 30 min	Admission
99222	Initial hospital care moderate 50 min	Admission
99223	Initial hospital care high 70 min	Admission
99231	Subsequent hospital care sf/low 15 min	Office visit
99232	Subsequent hospital care moderate 25 min	Office visit
99233	Subsequent hospital care high 35 min	Office visit
99238	Hospital discharge day, 30 min or less	Admission
99239	Hospital discharge day, more than 30 min	Admission
99241	Office o/p consultation sf 15 min	Office visit
99242	Office o/p consultation sf 30 min	Office visit

© 2026 O'Meara D et al. *JAMA Netw Open*.

99243	Office o/p consultation low 40 min	Office visit
99244	Office o/p consultation moderate 60 min	Office visit
99245	Office o/p consultation high 80 min	Office visit
99251	Inpatient consultation sf 20 min	Inpatient consult
99252	Inpatient consultation sf 40 min	Inpatient consult
99253	Inpatient consultation low 55 min	Inpatient consult
99254	Inpatient consultation moderate 80 min	Inpatient consult
99255	Inpatient consultation high 110 min	Inpatient consult
99281	Emergency dept visit sf	ED visit
99282	Emergency dept visit low	ED visit
99283	Emergency dept visit moderate (history and exam is expanded problem focused)	ED visit
99284	Emergency dept visit moderate (history and exam is detailed)	ED visit
99285	Emergency dept visit high	ED visit
95819	EEG: *Awake and Asleep*	EEG
95812	EEG: Extended 41-60 min	EEG
95813	EEG: Extended 61-119 min	EEG
95816	EEG: *Awake and Drowsy*	EEG
95819	EEG: PORT *Awake and Asleep*	EEG
95812	EEG: PORT Extended 41-60 min	EEG
95813	EEG: PORT Extended 61-119 min	EEG
95718	VEEG 2-12 hrs Continuous	EEG
95957	EEG: Digital Analysis/SP-SZ Detection	EEG
95829	Electrocorticogram Surg (G&S MoyaMoya)	EEG
95961	Functional Cortical	EEG
	(stimulation): Initial hour	
95962	Functional Cortical (stimulation): ______ Hrs	EEG
95958	WADA Activation	EEG
95718	VEEG 2-12 hrs Intermittent	EEG
95720	VEEG 12-26 hrs Intermittent	EEG
95718	VEEG 2-12 hrs Unmonitored	EEG
95720	VEEG 12-26 hrs Unmonitored	EEG
95717	EEG 2-12 hrs Unmonitored (w/o video)	EEG
95719	EEG 12-26 hrs Unmonitored (w/o video)	EEG
95719	EEG 26-36 hrs Unmonitored (w/o video)	EEG

95717		EEG
95721	EEG 36-50 hrs Unmonitored (w/o video)	EEG
95721	EEG 50-60 hrs Unmonitored (w/o video)	EEG
		EEG
95723	EEG 60-74 hrs Unmonitored (w/o video)	EEG
95723	EEG 74-84 hrs Unmonitored (w/o video)	EEG
		EEG
95725	EEG 84-98 hrs Unmonitored (w/o video)	EEG
95860	EMG - 1 extremity	EMG/NCS
95861	EMG - 2 extremities	EMG/NCS
95872	EMG - Needle EMG Single Fiber	EMG/NCS
95870	EMG - Needle limited study of muscles	EMG/NCS
	_____ Units (max 4)	EMG/NCS
95867	EMG - Cranial N, unilateral	EMG/NCS
95868	EMG - Cranial N, bilateral	EMG/NCS
95885	EMG - each extrem. w/ nerve conduction; limited _____(Max 4)	EMG/NCS
		EMG/NCS
95886	EMG - each extrem. w/ nerve conduction; complete____ (Max 4)	EMG/NCS
		EMG/NCS
95887	EMG - non-extrem. (cranial nerve) w/ nerve conduction	EMG/NCS
95865	EMG - Needle Electromyography Larynx	EMG/NCS
95907	1-2 nerve conduction studies	EMG/NCS
95908	3-4 nerve conduction studies	EMG/NCS
95909	5-6 nerve conduction studies	EMG/NCS
95910	7-8 nerve conduction studies	EMG/NCS
95911	9-10 nerve conduction studies	EMG/NCS
95912	11-12 nerve conduction studies	EMG/NCS
95913	13+ nerve conduction studies	EMG/NCS
95937	Neuromusc. Junct. test __ per nerve	EMG/NCS
93886	Transcranial Doppler study of intracranial arteries, complete	Transcranial Doppler

eTable 4. Single Ventricle Procedure and Diagnosis Codes

	**ICD-9**	**ICD-10**	**CPT**	**ICD-10 cleaned**
Procedure				Procedure Codes
Fontan	35.94 (Creation of conduit between atrium-PA)	PCS codes for inpatient - 06100JP Fontan to pulmonary trunk, 06100JQ Fontan to RPA, 06100JR Fontan to LPA	33615, 33617	06100JP
Norwood	[35.41 or 35.42] (Creation or enlargement of septal defect) + [39.0 or 35.92] (Systemic-PA shunt or creation of RV-PA conduit) + [Any of 38.35, 38.45, 38.34, 38.44, 38.64, 38.65, 38.84, 38.85, 39.56, 39.57, 39.58, 39.59, 36.99] (resection/excision/occlusion)	PCS codes for inpatient - 02UX07Z Arch repair with autologous tissue, 02UX0JZ Arch repair with synthetic substitute, 021KOJP RV to PA conduit to pulmonary trunk with synthetic substitute, 021K0JQ RV to PA conduit to RPA with synthetic substitute, 021K0JR RV to PA conduit to LPA with synthetic substitute, 02B50ZZ Atrial septectomy and then one of the DKS codes below	33619	02UX07Z
Damus-Kay-Stansel		PCS codes for inpatient - 021X08P DKS Zooplastic tissue, 021X0JP DKS Synthetic substitute, 021X0KP DKS Non autologous tissue, 021X0ZP DKS Direct anastomosis	33606	021X08P
Glenn	39.21 (Caval-pulmonary artery anastomosis)	PCS codes for inpatient - 021VOZP SVC to pulmonary trunk, 021V0ZQ SVC to RPA, 021V0ZR SVC to LPA	33766, 33767, 33768	021VOZP
Diagnosis				**Diagnosis Codes**
Hypoplastic left ventricle	745.8	Q20.8		Q20.8
Hypoplastic right ventricle	745.8	Q20.8		Q20.8
Hypoplastic left heart syndrome	746.7	Q23.4		Q23.4
Hypoplastic right heart syndrome	746.1	Q22.6		Q22.6

© 2026 O'Meara D et al. *JAMA Netw Open*.

DIRV	745.3	Q20.4		Q20.4
DILV	745.3	Q20.4		Q20.4
DOLV	745.19	Q20.2		Q20.2
DORV	745.11	Q20.1		Q20.1
Other cardiac septa	745.8	Q21.8 other congenital malformation of cardiac septa		Q21.8
Other cardiac septa	745.9	Q21.9 Congenital malformation of cardiac septa unspecified		Q21.9

eTable 5. Neurodevelopmental Diagnoses at Any Time Until 5 Years Following Index Surgery (Not Accounting for Variable Follow-Up)

**Neurodevelopmental Disorder Categories**	**N (%)**
ADHD	32 (1)
Autism	84 (2.7)
Communication disorder	791 (25.1)
Global developmental delay	482 (15.3)
Intellectual disability	67 (2.1)
Learning Disorder	4 (0.1)
Motor Disorder	585 (18.6)
Other neurodevelelopmental disorder	106 (3.4)
Tic disorder	2 (0.1)
Any	*1278 (40.6)*

© 2026 O'Meara D et al. *JAMA Netw Open*.

eTable 6. Behavioral Diagnoses at Any Time Until 5 Years Following Index Surgery (Not Accounting for Variable Follow-Up)

**Behavioral Disorder Categories**	**N (%)**
Anxiety Disorders	71 (2.3)
Depressive Disorders	9 (0.3)
Disruptive and Impulse Control Disorders	47 (1.5)
Elimination Disorders	24 (0.8)
Feeding and Eating Disorders	122 (3.9)
Mental Health Symptom	253 (8)
Miscellaneous	57 (1.8)
Obsessive Compulsive and Related Disorders	18 (0.6)
Sleep Wake Disorders	26 (0.8)
Somatic Symptom and Related Disorders	8 (0.3)
Substance Related and Addictive Disorders	166 (5.3)
Trauma and Stressor Related Disorders	99 (3.1)
Any	*674 (21.4)*

© 2026 O’Meara D et al. *JAMA Netw Open*.

eTable 7. Neurological Diagnoses at Any Time Until 5 Years Following Index Surgery (Not Accounting for Variable Follow-Up)

**Neurological Categories**	**N (%)**
**Genetic**	**709 (22.5)**
Down syndrome	430 (13.7)
DiGeorge syndrome	153 (4.9)
**Brain and spinal cord abnormalities**	**434 (13.8)**
Microcephaly	119 (3.8)
Brain malformation	126 (4.0)
**Epilepsy**	**223 (7.1)**
**Muscular dystrophy or myopathy**	**202 (6.4)**
Congenital Hypotonia	182 (5.8)
**Cerebral palsy or hypoxic ischemic encephalopathy**	**124 (3.9)**
**Cerebrovascular**	**112 (3.6)**
**Encephalopathy**	**90 (2.9)**
**Plegia or paralysis**	**73 (2.3)**
**Hydrocephalus**	**63 (2.0)**
**Any**	**1346 (42.8)**

© 2026 O'Meara D et al. *JAMA Netw Open*.
