# Supplementary material for: Neurodevelopment Among Publicly Insured Children in the First 5 Years After Infant Heart Surgery
Source: JAMA Netw Open. 2026 Feb 4;9(2):e2556832. doi: 10.1001/jamanetworkopen.2025.56832 (PMC12873768; doi:10.1001/jamanetworkopen.2025.56832)
Supplement: Supplement 2. — Data Sharing Statement [file jamanetwopen-e2556832-s002.pdf]

Data Sharing Statement

O'Meara. Neurodevelopment Among Publicly Insured Children in the First 5 Years After Infant Heart Surgery. *JAMA Netw Open*. Published February 02, 2026.
doi:10.1001/jamanetworkopen.2025.56832

Data
Data available: No

Additional Information
Explanation for why data not available: Merative data (specifically the Merative MarketScan Research Databases) are proprietary, so we are not allowed to give others access to our data. However, data are available directly to others from Merative for a fee or with an institutional agreement.
